# Supplementary material for: Evaluation of a complex intervention (Engager) for prisoners with common mental health problems, near to and after release: study protocol for a randomised controlled trial
Source: BMJ Open. 2018 Feb 20;8(2):e017931. doi: 10.1136/bmjopen-2017-017931 (PMC5879493; doi:10.1136/bmjopen-2017-017931)
Supplement: Supplementary file 1 [file bmjopen-2017-017931supp001.pdf]

Appendix 1

PLYMOUTH UNIVERSITY PENINSULA SCHOOLS OF MEDICINE & DENTISTRY

engager healthy minds, healthy choices

MANCHESTER 1824 The University of Manchester

Information for people considering taking part in the Engager Randomised Controlled Trial

What is the purpose of the study?

We have put together a package of care that aims to help people in prison and after they are released from prison. The purpose of this study is to find out how well this package of care works.

Why have I been asked to take part?

We are interested in talking to prisoners near release who may be experiencing anxiety, distress or feeling low.

Do I have to take part?

No, it is up to you. If you would prefer not to take part then you do not have to give a reason and you will not be under any pressure to change your mind, and this decision will not affect the normal help and services you receive when leaving prison. If you do decide to take part you will be asked to sign a consent form to show you have agreed to take part. If you do decide to take part then you are free to leave the study at any time. You do not have to give a reason but any information you have already given will remain part of the research and the research team may contact you to ask if there are ways in which you think the service could be improved.

What does taking part involve?

If you agree to take part, we will first ask you some questions about how you have been feeling over recent weeks and about any problems you had before you came into prison. This should take about 20 minutes and will help us make a decision about whether the intervention is suitable for you. If the intervention is not suitable for you then we will not need to see you again and that will be the end of your participation in the study. This does not mean that your problems are more or less important than anyone else's problems; it just means that our intervention is not suitable for you.

If the intervention it suitable for you then we would like to ask you further questions about how you have been feeling recently and what sort of things are likely to be a problem for you when you get released. This should take about 1 hour and we can do it immediately after the first set of questions or we can arrange another time for me to come back and see you.

Once you have finished these questions, you will be put in either the Engager Intervention group or the Treatment As Usual group. A computer programme will randomly put you in one of the two groups. If you are not put in the Engager intervention group it does not mean that your needs are any more or less important.

Treatment as usual

If you are in the Treatment As Usual group you will be involved in the standard discharge planning within the prison, nothing different will happen to you and you will receive all the services that you would normally receive leading up to and following your release from prison.

Engager Intervention

If you are in the Engager Intervention group you will receive all the standard discharge planning that you would normally receive, but you will also work with the Engager Intervention team. While in prison they will work with you to look at what your needs might be on release from prison and develop a tailor-made package of care for you. This will include supporting you in managing your anxiety and mood, and looking at your goals and concerns about

release which might include housing, finances, relationships, and health and wellbeing. They will also explore any community services that might be able to help you when you are released from prison.

The Engager Intervention team will continue to work with you through the gate and for up to four months following your release to support you. They will be in contact with you between 10 and 20 times in the three months following your release from prison, depending on your needs and how well things are going for you. Some of these contacts will be face-to-face, but others may be by phone. We may want to record one or more of your meetings with the Engager Intervention team for training purposes. The Engager Intervention team will inform you at the start of any meeting that they would like to record it and seek your written permission to do so. It is entirely up to you whether you give permission for any of your meetings to be recorded. If you do not want to have any meetings recorded, this will not affect your participation in other aspects of the study.

We will ask for your permission for the Engager Intervention team to access your criminal justice and health records in order to help them develop a tailor-made package of care with you. We will also ask your permission to share the questionnaires you have just completed with them.

The Engager team may also need to share your tailor-made care plan with other organisations that will be involved in helping you.

Will I have to do anything else?

Regardless of whether you are allocated to the 'Engager Intervention' group or 'Treatment As Usual' group, a member of the research team would like to stay in touch with and meet with you to ask you some questions about how well you have been getting on. The questions will be about your health needs, use of services and how you have been feeling. This will be around a week before you are released and at 4 weeks, 3 months, 6 months, and 12 months following your release from prison. We will speak to you about this nearer the time. As a thank you for staying in touch you will receive high-street vouchers at the end of the 3, 6, and 12 months sessions, although you will only receive these if we meet you in the community and not if you are in prison at the time.

We will ask for your permission for the Engager Research team to access your criminal justice and health records in order to inform the research. Information will be collected after your session with the researcher today and then again in 12 months' time from the Police National Computer to see if any of this information has changed.

While you are in prison and after your release, you may also be invited to an interview with a researcher to discuss your thoughts about any treatment you may have received, and your experience of being involved in the study. Similarly, if you decide not to take part in the Randomised Controlled Trial you may be invited to be interviewed in order to help us to understand the reasons why you don't want to be involved. It is entirely up to you to decide whether you want to take part in further parts of the study and by agreeing to take part now you are not committing yourself to being involved in any other parts of the study.

What are the possible benefits of taking part?

We can't promise that the study will help you directly, but we hope that for those in the Engager Intervention group taking part in the study may help you to manage your anxieties and mood more effectively and improve the contact you have with services after release from prison. Findings from the research may also help to improve services for future prisoners when they are released from prison.

What are the possible risks or disadvantages of taking part?

You will be asked to give up some of your time to take part. We do not see any serious risks in taking part in the study. Occasionally some people may experience some emotional distress when they are asked to think about their experiences. If you would prefer not to answer any individual questions just say so and we will more onto the next

question. If you are upset you will be able to talk to the researcher about it. If you feel you require further support they will be able to tell you about other possible sources of help or advice.

What if there is a problem?

If you are in prison or under probation supervision then you should direct any requests for information, complaints, concerns and queries through the prison establishment or the probation service. If you are not in prison or under probation supervision and have a problem or concern about the way you have been approached or treated during this study then you should ask to speak to the researcher, who will do their best to answer your questions. In the event that something goes wrong and you are harmed during the research, and this is due to someone's negligence, then you may have grounds for a legal action for compensation but you may have to pay your own legal costs.

Will my taking part in the study be kept confidential?

Yes. Any information collected about you will be kept strictly confidential and will not be disclosed outside the research team without your permission. Any personal information that we collect about you, and any consent forms will be stored securely and will only be used for the purposes of the research. Transcription of audio recordings of any meetings between yourself and the Engager team will only be done by members of the research team or other individuals who have signed confidentiality agreements. Any quotations from research participants used in the project report will be anonymised and no real names will be used.

You have the right to check the accuracy of the data held about you and to correct any errors. Procedures for the handling, processing, storage and destruction of your data will be compliant with the Data Protection Act 1998. Some parts of the data collected for the study may be looked at by authorised representatives of regulatory authorities to check that the study is being correctly carried out, but this information will remain anonymous. Should this occur, all such individuals will have a duty of confidentiality to you as a research participant. The data will be securely disposed of after 5 years.

Are there any circumstances in which confidentiality would be broken?

Yes. You should be aware that the researcher has a duty to inform an appropriate person should you disclose any of the following:

a) Behaviour that is against prison rules and can be adjudicated against
b) Information that indicates a risk of harm to yourself or others or refers to a new crime committed or plans to commit a new crime
c) Undisclosed illegal acts
d) Information that raises concerns about terrorism, radicalisations, or security issues.

What will happen to the results of the study?

The results of this study may be published in a report or criminal justice or medical journal. If you would like a copy of any publication or a summary of the results, please let the researcher know. You will not be identified in any report or publication arising from the study.

Who will know if I am taking part in this study?

The research team and practitioners delivering the intervention will know you are taking part in the study. Staff from the prison service may also be aware that you are taking part in the research, but they will not have access to any information we collect during the course of the study.

Who has reviewed this study?

The research has been looked at by an independent group of people, called a Research Ethics Committee (REC) to protect your safety, rights, wellbeing and dignity. This study has been reviewed and given a favourable opinion by NRES Committee Wales REC 3. The research has also been reviewed and approved by the Research & Development Offices of your local NHS Trust and NOMS.
