# Supplementary material for: Evaluation of a complex intervention (Engager) for prisoners with common mental health problems, near to and after release: study protocol for a randomised controlled trial
Source: BMJ Open. 2018 Feb 20;8(2):e017931. doi: 10.1136/bmjopen-2017-017931 (PMC5879493; doi:10.1136/bmjopen-2017-017931)
Supplement: Supplementary file 2 [file bmjopen-2017-017931supp002.pdf]

Appendix 2

PLYMOUTH UNIVERSITY PENINSULA
SCHOOLS OF MEDICINE & DENTISTRY

engager
healthy minds, healthy choices

MANCHESTER 1824
The University of Manchester

CONSENT FORM

*Please **initial***

1. I confirm that I have read and understood the research information sheet ('Information for people considering taking part in the Engager Randomised Controlled Trial') dated 24/03/2017, Version Five, and have been given the opportunity to ask questions. ☐
2. I understand that my participation is voluntary and that I am free to withdraw at any time, without giving any reason and without my legal rights being affected. ☐
3. I understand that if I withdraw from the study I will stop receiving the intervention, but this will not affect any other aspects of my medical care. ☐
4. I understand that whilst I participate in the RCT, the researcher has a duty to inform prison staff should I disclose:
   a) Behaviour that is against prison rules and can be adjudicated against
   b) Information that either indicates a risk or harm to yourself or others or refers to a new crime committed or plans to commit a new crime ☐
   c) Undisclosed illegal acts
   d) Information that raises concerns about terrorist, radicalisations, or security issues.
5. I understand that I will be assigned to the intervention group or treatment as usual group at random, and I won't be able to choose which group I am in. ☐
6. I understand that I may be asked for permission to digitally record one or more of my meetings with the Engager team, but I am under no obligation to give permission. ☐
7. I give permission for the research team to look at my criminal justice and health records for the purpose of informing the research. ☐
8. I give the research team permission to request my criminal justice records from the Police National Computer in 12 months' time ☐
9. I agree to take part in the above study. ☐
10. I do not wish to take part in the study but agree to be contacted to discuss my reasons for not wanting to be involved. ☐
11. I understand that I may be contacted by the research team in the future, but am under no obligation to participate in any further research. ☐

Sign below for participant completed consent form

Name of participant	Date	Signature
Name of researcher	Date	Signature

p.t.o. for researcher completed consent forms

PLYMOUTH UNIVERSITY PENINSULA SCHOOLS OF MEDICINE & DENTISTRY

engager
healthy minds, healthy choices

MANCHESTER 1824
The University of Manchester

Sign below for researcher completed consent form

"I [researcher name] have read this form of consent to [participant name] because [participant name] is not able to read this informed consent document.

I have asked [participant name] to make his mark to confirm that he has understood the informed consent document"

Name of participant	Date	Signature
Name of researcher	Date	Signature

*1 copy for participant: 1 copy for researcher

Principal Investigator: Professor Richard Byng, Plymouth University Peninsula Schools of Medicine and Dentistry
