# Supplementary material for: Evaluation of a complex intervention (Engager) for prisoners with common mental health problems, near to and after release: study protocol for a randomised controlled trial
Source: BMJ Open. 2018 Feb 20;8(2):e017931. doi: 10.1136/bmjopen-2017-017931 (PMC5879493; doi:10.1136/bmjopen-2017-017931)
Supplement: Author's manuscript [file bmjopen-2017-017931.draft_revisions.pdf]

BMJ Open

BMJ Open is committed to open peer review. As part of this commitment we make the peer review history of every article we publish publicly available.

When an article is published we post the peer reviewers' comments and the authors' responses online. We also post the versions of the paper that were used during peer review. These are the versions that the peer review comments apply to.

The versions of the paper that follow are the versions that were submitted during the peer review process. They are not the versions of record or the final published versions. They should not be cited or distributed as the published version of this manuscript.

BMJ Open is an open access journal and the full, final, typeset and author-corrected version of record of the manuscript is available on our site with no access controls, subscription charges or pay-per-view fees (http://bmjopen.bmj.com).

If you have any questions on BMJ Open's open peer review process please email editorial.bmjopen@bmj.com

BMJ Open

Evaluation of a complex intervention (Engager) for prisoners with common mental health problems, near to and after release – study protocol for a randomised controlled trial.

Journal:	*BMJ Open*
Manuscript ID	bmjopen-2017-017931
Article Type:	Protocol
Date Submitted by the Author:	06-Jun-2017

Complete List of Authors:	Kirkpatrick, Tim; Plymouth University, Peninsula Schools of Medicine and Dentistry<br>Lennox, Charlotte; University of Manchester, Manchester Academic Health Science Centre<br>Taylor, Rod; University of Exeter, Exeter Medical School<br>Anderson, Rob; University of Exeter, Peninsula Technology Assessment Group (PenTAG)<br>Maguire, Michael; University of South Wales, Centre for Criminology<br>Haddad, Mark; Centre for Mental Health Research, Professor of Criminology<br>Michie, Susan; University College London, Centre for Outcomes Research and Effectivenes<br>Owens, Christabel ; University of Exeter Medical School , Institute of Health Research<br>Durcan, Graham; Centre for Mental Health<br>Stirzaker, Alex; Avon and Wiltshire Mental Health Partnership NHS Trust, Associate Director for Criminal Justice Programme<br>Henley, W; Institute of Health Research, University of Exeter Medical School, College House, St Luke's Campus, Exeter, Devon, UK, EX1 2LU<br>Stevenson, Caroline; The University of Manchester<br>Carroll, Lauren; Plymouth University Peninsula Schools of Medicine and Dentistry<br>Quinn, Cath; Plymouth University Peninsula Schools of Medicine and Dentistry, Primary Care, Clinical Trials and Population Studies<br>Brand, Sarah; Plymouth University Peninsula Schools of Medicine and Dentistry<br>Harris, Tirril; Kings College, University of London, Socio-Medical Research Group, Institute of Psychiatry<br>Stewart, Amy; Plymouth University Peninsula Schools of Medicine and Dentistry, Primary Care, Clinical Trials and Population Studies<br>Todd, Roxanne; The University of Manchester, Division of Psychology & Mental Health<br>Rybczynska-Bunt, Sarah; Plymouth University Peninsula Schools of Medicine and Dentistry, Primary Care, Clinical Trials and Population Studies<br>Greer, Rebecca; Plymouth University Peninsula Schools of Medicine and Dentistry, Primary Care, Clinical Trials and Population Studies<br>Pearson, Mark; University of Exeter Medical School, University of Exeter, Peninsula Technology Assessment Group (PenTAG)<br>Shaw, Jenny; University of Manchester, Academic Health Science Centre<br>Byng, Richard; Plymouth University Peninsula Schools of Medicine and

	Dentistry, Primary Care, Clinical Trials and Population Studies
**Primary Subject Heading**:	Mental health
Secondary Subject Heading:	Health services research
Keywords:	Prison, Offender, Randomised controlled trial, MENTAL HEALTH

SCHOLARONE™ Manuscripts

For peer review only

Evaluation of a complex intervention (Engager) for prisoners with common mental health problems, near to and after release – study protocol for a randomised controlled trial.

T Kirkpatrick, C Lennox, RS Taylor, R Anderson, M Maguire, M Haddad, S Michie, C Owens, G Durcan, A Stirzaker, W Henley, C Stevenson, L Carroll, C Quinn, SL Brand, T Harris, A Stewart, R Todd, S Rybczynska-Bunt, R Greer, M Pearson, J Shaw, R Byng

Corresponding author:
Dr T Kirkpatrick:
Plymouth University Peninsula Schools of Medicine and Dentistry,
N9 Plymouth Science Park,
Davy Road,
Derriford,
Plymouth,
PL6 8BX
Email: tim.kirkpatrick@plymouth.ac.uk
Phone: 01752 764477
Fax: 01752 764234

Co-authors:
Division of Psychology and Mental Health, 2.315 Jean McFarlane Building, The University of Manchester, Oxford Road, Manchester, M13 9PL C Lennox, Research Fellow and Lecturer University of Exeter Medical School, St Luke's Campus, Heavitree Road, Exeter EX1 2LU RS Taylor Chair of Health Services Research Exeter Medical School, St Luke's Campus, Heavitree Road, Exeter EX1 2LU R Anderson Associate Professor Centre for Criminology, University of South Wales, Pontypridd, CF37 1DL M Maguire Professor of Criminology Centre for Mental Health Research, School of Health Sciences, City, University of London, Northampton Square, London EC1V 0HB M Haddad Senior Lecturer UCL Centre for Behaviour Change, University College London, 1-19 Torrington Place, London WC1E 7HB S Michie Director of UCL Centre for Behaviour Change University of Exeter Medical School, St Luke's Campus, Heavitree Road, Exeter EX1 2LU C Owens Associate Professor Centre for Mental Health, Unit 2D21 Technopark, LSBU 90 London Road, London SE1 6LN G Durcan Associate Director for Criminal Justice Program Avon and Wiltshire Mental Health Partnership NHS Trust, LIFT Psychology, Old Town Surgery, Curie Avenue, Swindon SN1 4GB A Strizaker Clinical Psychologist University of Exeter Medical School, St Luke's Campus, Heavitree Road, Exeter EX1 2LU W Henley Professor of Medical Statistics Division of Psychology and Mental Health, 2.315 Jean McFarlane Building, The University of Manchester, Oxford Road, Manchester, M13 9PL C Stevenson Research Assistant Plymouth University Peninsula Schools of Medicine and Dentistry, N9 Plymouth Science Park, Davy Road, Derriford, Plymouth, PL6 8BX L Carroll Research Assistant Plymouth University Peninsula Schools of Medicine and Dentistry, N9 Plymouth Science Park, Davy Road, Derriford, Plymouth, PL6 8BX C Quinn Senior Research Fellow Plymouth University Peninsula Schools of Medicine and Dentistry, N9 Plymouth Science Park, Davy Road, Derriford, Plymouth, PL6 8BX SL Brand Research Fellow Socio-medical Research Group, Institute of

For peer review only - http://bmjopen.bmj.com/site/about/guidelines.xhtml

Psychiatry, Kings College, University of London T Harris Honorary Research Fellow Plymouth University Peninsula Schools of Medicine and Dentistry, N9 Plymouth Science Park, Davy Road, Derriford, Plymouth, PL6 8BX A Stewart Research Assistant Division of Psychology and Mental Health, 2.315 Jean McFarlane Building, The University of Manchester, Oxford Road, Manchester, M13 9PL R Todd Research Assistant Plymouth University Peninsula Schools of Medicine and Dentistry, N9 Plymouth Science Park, Davy Road, Derriford, Plymouth, PL6 8BX S Rybczynska-Bunt Research Fellow Plymouth University Peninsula Schools of Medicine and Dentistry, N9 Plymouth Science Park, Davy Road, Derriford, Plymouth, PL6 8BX R Greer Research Assistant University of Exeter Medical School, St Luke's Campus, Heavitree Road, Exeter EX1 2LU M Pearson Senior Research Fellow Division of Psychology and Mental Health, 2.315 Jean McFarlane Building, The University of Manchester, Oxford Road, Manchester, M13 9PL J Shaw Professor of Forensic Psychiatry Plymouth University Peninsula Schools of Medicine and Dentistry, N9 Plymouth Science Park, Davy Road, Derriford, Plymouth, PL6 8BX R Byng Professor of Primary Care Research

Key words

Prison, offender, Randomised controlled trial, mental health, protocol

Word count: 5476 (+286 in abstract)

For peer review only

ABSTRACT

Introduction

The 'Engager' programme is a 'through-the-gate' intervention designed to support prisoners with common mental health problems as they transition from prison back into the community. The trial will evaluate the clinical and cost effectiveness of the Engager intervention.

Methods and Analysis

The study is a parallel two-group randomised controlled trial (RCT) with 1:1 individual allocation to either: a) the Engager intervention plus standard care (intervention group), or b) standard care alone (control group) across two investigation centres (South West and North West of England). Two hundred and eighty offenders meeting eligibility criteria will take part. Engager is a person-centred complex intervention delivered by practitioners comprising one-to-one support for offenders prior to release from prison and for up to 20 weeks post release. It aims to address offenders' mental health and social care needs. The primary outcome is change in psychological distress measured by the Clinical Outcomes in Routine Evaluation – Outcome Measure (CORE-OM) at six months post-release. Secondary outcomes include: assessment of subjective met/unmet need, drug and alcohol use, health related quality of life, and wellbeing related quality of life measured at three and six months post release; change in objective social domains, drug and alcohol dependence, service utilisation, perceived helpfulness and change in trust, hope and motivation at six months post release; and recidivism at 12 months post release. A process evaluation will assess fidelity of intervention delivery, test hypothesised mechanisms of action and look for unintended consequences. An economic evaluation will estimate the cost-effectiveness.

Ethics and Dissemination

This study has been approved by the Wales Research Ethics Committee 3 (ref: 15/WA/0314) and the National Offender Management Service (NOMS; ref: 2015-283). Findings will be disseminated to commissioners, clinicians and service users via papers and presentations.

Trial registration: ISRCTN11707331. Registration date 04/02/2016

[286 words, excluding trial registration details]

Strengths and limitations of this study

- The study will be a two-centre, randomised controlled trial of a through-the-gate intervention for offenders with common mental health problems; it will provide much needed evidence of what works for this difficult to engage population.
- The primary and secondary outcomes have been selected following extensive piloting work and cover a broad range of outcome domains that could be impacted by the complex intervention.
- The study adopts a flexible and pragmatic approach to data collection to try to overcome the challenges of following up this population after release from prison.
- The lack of blinding of researchers collecting study data is a limitation of the study design.

For peer review only - http://bmjopen.bmj.com/site/about/guidelines.xhtml

For peer review only

INTRODUCTION

People in contact with all stages of the Criminal Justice System (CJS), but especially prisoners, have a high prevalence of mental health problems and yet trials in this setting are limited. Rates of 50-90% have been found among prison populations, both in the UK,[1,3] and internationally.[4] In England and Wales, an Office of National Statistics survey reported high rates of personality disorder (PD) (64%), neurotic disorders (40%), drug dependency (43%) and hazardous alcohol use (63%) in sentenced prisoners, with higher rates generally being found in remand prisoners.[5] Post-traumatic stress disorder (PTSD) was also reported to be prevalent within the prison population. High levels of self-harming behaviour and suicidal thoughts have also been reported, with the risk of suicide for male offenders leaving prison being eight times the national average.[6] Our development work indicated high rates of anxiety and depression, with 47% reaching likely caseness for anxiety, PTSD or depression while in prison, of which 32% were still 'cases' after release. There was also substantial co-morbidity.[7]

In addition to mental health problems, offenders have wide-ranging personal and social problems, including homelessness, unemployment, broken relationships with both partners and children, and they typically live chaotic lives. In our previous study, 37% reported problems with family relationships; the majority of the sample were unemployed or on long-term sickness benefit (65% in prison and 70% in the community sample), and 26% had on-going legal or criminal justice issues.[8] These results echo previous surveys of prisoners.[9-12] The Surveying Prisoner Crime Reduction (SPCR) study, which is based on regular interviews with a cohort of prisoners before and after release, shows two-thirds reporting unemployed status before going into custody, and 37% reporting the need for assistance in finding accommodation on release. [13] These issues tend to be the focus of offenders' own concerns, indicating a crucial need to address such issues, whilst providing motivation for change.

The cost of failing to address these issues is high. Those serving short-term sentences place a considerable burden on society. Twelve-month proven re-offending rates for short-sentence prisoners are currently close to 60% [14] and, in addition to the distress and inconvenience commonly experienced by their victims, many 'volume' offences have a surprisingly high financial impact. For example, in 2010 the costs of an average domestic burglary were estimated at £3,925 and of a less serious wounding at £9,790.[15] Therefore, the potential benefits of addressing these issues, to individuals and communities, as well the financial savings, are significant.

There are complex relationships between mental health, substance misuse, social exclusion and criminal behaviour. However, these tend to be studied separately, with interventions designed to address them being developed and delivered in isolation. An underpinning aim of the Engager intervention is to identify and overcome service barriers, particularly between health and criminal justice sectors, and embed multi-agency working within the intervention.

Prison healthcare is often provided by a number of different NHS, private, or third sector organisations each providing separate primary care, mental health care, drug and alcohol services. Opiate substitution services are now generally available in prison, and mechanisms for achieving continuity post-release are improving. Mental health services for those with severe and enduring mental illness have faced considerable challenges,[16] but have improved care for those with psychosis, with new evidence now supporting the development of mental health pathways on release.[17]

For peer review only - http://bmjopen.bmj.com/site/about/guidelines.xhtml

Provision of psychological therapy for offenders with common mental health problems is limited in both prison and community settings.[8] The prison environment complicates diagnostic assessment,[3] and for some fewer stressors in prison may reduce anxiety, making it difficult to identify mental health problems that may arise post-release. The focus of hard-pressed prison healthcare staff is on immediate concerns, rather than longer-term post-release planning. Improving Access to Psychological Therapies (IAPT) services in prisons are still in the early stages of development.

Once released into the community, ex-prisoners with common mental health problems are, in theory, provided for by mainstream statutory services including general practice, community mental health teams and IAPT services. In reality, few access these services. For example, in a previous study we found an average of only 0.96 contacts with mental health services per offender per year for those reporting common mental health problems,[9] suggesting that a lack of care on release is the norm.[18]

Despite negligible uptake and high need, no systems worldwide have been identified for actively engaging offenders with common mental health problems whilst in prison, providing initial treatment and transferring care to community teams. Many offenders, like others with common mental health problems complicated by co-morbidity, fall between primary care, IAPT and specialist services[19-22]. Offenders are further disadvantaged by their reluctance both to seek help and to accept mental health diagnoses, and by lower levels of GP registration.[7,8,18] Services can also be seen as resistant to offenders, and are not designed to meet the needs of those with complex and multiple vulnerabilities.[23] This contrasts with well-established services, together with arrangements for transfer of care, for those with opiate misuse.[24,25] In a relatively small proportion of cases, psychological input and/or general support is provided by statutory or third-sector resettlement services, from probation-delivered thinking skills 'booster' programmes for prisoners on licence, through to volunteer or peer-mentoring services,[26,27] although currently resettlement plans typically contain limited reference to health concerns.

In view of these factors, provision of care for common mental health problems should be considered as part of the range of services making up collaborative care and directed towards improving social outcomes and resettlement. The Engager research programme was designed to develop and evaluate a collaborative care intervention for prisoners with common mental health problems, near to and after release from prison, supporting multiple needs rather than focused on specific diagnoses or on a particular therapy. We describe the methods of the Engager trial here.

AIMS AND HYPOTHESIS

The Engager trial aims to answer the research question: *What is the effectiveness and cost-effectiveness of the Engager Intervention plus usual care, compared to usual care alone, in prisoners with common mental health problems, both before release and up to 20 weeks following release from prison?* The primary hypothesis is that the participants receiving the Engager intervention plus usual care (the 'intervention group') will have reduced levels of psychological distress as measured by the Clinical Outcomes in Routine Evaluation – Outcome Measure (CORE-OM)[28] at six months post-release from prison (primary outcome) compared to participants receiving usual care alone (the 'control group'). Secondary hypotheses of the trial are that, compared to the control group, the intervention group will have:

- an increase in the number of subjective met needs and decrease in the number of unmet needs in relation to accommodation, education, work/money/benefits, family/friends/company/intimacy, physical and mental health, safety to self, and self-care, safety to others, and leisure activities;

For peer review only - http://bmjopen.bmj.com/site/about/guidelines.xhtml

- a decrease in substance use and subjective view of dependence;
- improvement in generic health related quality of life, wellbeing related quality of life, subjective experience of care received and perceived helpfulness of services ;
- a reduction in levels of proven re-offending;

The trial will estimate the total cost, and per prisoner cost, of providing the Engager intervention, and the cost-effectiveness of the Engager intervention plus usual care versus usual care alone across health, social care, and criminal justice sectors.

The trial also includes a parallel process evaluation, designed to: determine the degree to which the core mechanisms of the intervention were delivered; evaluate the extent to which the core mechanisms of the intervention produced the intended outcomes; identify aspects of the intervention and delivery that could be improved, and explore unintended consequences of the intervention.

METHODS AND ANALYSIS

Design

The study is a parallel two-group randomised controlled trial (RCT) with 1:1 individual participant allocation to either the Engager Intervention plus standard care (intervention group) or standard care alone (control group), with economic evaluation and parallel process evaluation.

Setting

The study will be conducted in two investigation centres (South West and North West of England). Participants will be recruited from three prisons, two in the South West and one in the North West of England. Participants will be recruited in equal numbers from each of the two investigation centres, for both the intervention and control groups. Recruitment and baseline interviews will take place in the prisons, with follow-up interviews taking place in a suitable community location or (for those who are back in prison) in prison. Conduct of the trial in each centre will be led by a local Principal Investigator, supported by a research team, all of whom have received training in Good Clinical Practice and the requirements of the study protocol.

Study population

Potential participants will be men serving a custodial sentence of two years or less, who are within 4–20 weeks from release from prison and who are being released to the geographical area of the study. Potential participants will be identified using the Prison National Offender Management Information System (PNOMIS). Female prisoners, men on remand, and those with a diagnosis of serious mental illness or on the Offender Personality Disorder Pathway will be excluded from the trial. The full list of participant inclusion and exclusion criteria is provided in Table 1.

Individuals will be approached up to 20 weeks prior to release. Initial contact will be made by a member of the research team. They will be given the Participant Information Sheet and the researcher will discuss any queries/concerns with them. Researchers will take consent from individuals who wish to participate. All individuals providing written informed consent will complete a short screening interview to identify those

For peer review only - http://bmjopen.bmj.com/site/about/guidelines.xhtml

currently experiencing common mental health problems, or who have experienced common mental health problems in the previous two years that impacted on their day-to-day functioning, and are likely to experience similar problems on release. The screening interview comprises the Patient Health Questionnaire-9 (PHQ-9),[30] the Generalised Anxiety Disorder-7 (GAD-7),[31] the Primary Care PTSD Screen (PC-PTSD),[32] and a bespoke Historical Common Mental Health Problem screen. The researcher will read the questions to the participants, using a narrative conversational format developed in our pilot work to facilitate engagement. Individuals will be considered suitable for inclusion in the study if the screening interview indicates that they:

- Have a current common mental health problem as indicated by a score of 10 or more on the PHQ-9 or GAD-7, or 3 or more on the PC-PTSD;

or

- Have experienced a common mental health problem during the past two years which prevented them from functioning normally in everyday tasks, and which is likely to be a problem for them again following their release.

If a participant screens in following this assessment they will continue to the full baseline interview.

Table 1 – Trial entry criteria

Inclusion criteria
• Men with prison sentences of up to and including two years.<br>• Being released to the geographical area of the study.<br>• Having between 20 and 4 weeks remaining to serve in prison.<br>• Willing to engage with treatment services and research procedures.<br>• Identified using screening instruments as having, or likely to have following release, common mental health problems.
Exclusion criteria
• Men on remand.<br>• Women (numbers are smaller, and prisons are remote; resettlement needs are different; research procedures developed are not feasible for this context). Research will be in male prisons only.<br>• Those with serious and enduring mental disorder and/or on the caseload of the prison in-reach team.<br>• Those with active suicidal intent requiring management under the safer custody process or prison in-reach team, and where the healthcare team managing the prisoner feels it would be detrimental. Once risk levels reduce individuals in this group will be eligible if not excluded for another reason.<br>• Those with primary personality disorder who are on the caseload of the Offender Personality Disorder Pathway programme.<br>• Those who present a serious risk of harm to the researchers or intervention practitioners.<br>• Those unable to provide informed consent.

Participants will be informed that participation is voluntary and that they are free to withdraw from the study at any time, and it is stressed that withdrawal from the study will not affect their legal rights. They will also be informed that the researcher has a duty to inform prison staff if they disclose certain information, such as intent to harm self or others.

For peer review only - http://bmjopen.bmj.com/site/about/guidelines.xhtml

Randomisation

Participants will be individually randomised in a 1:1 ratio to receive either the Engager Intervention in addition to usual care, or usual care alone. To ensure concealment, randomisation will be achieved by means of a web-based system created by Peninsula Clinical Trials Unit (PenCTU). Randomisation numbers will be computer-generated and assigned in strict sequence. Randomisation will be stratified to ensure balance between the two treatment arms across the two investigator centres, with each centre having an independent sequence list for an equal number of participants. At the point of randomisation, participants will be assigned the next randomisation number in the sequence.

Confirmation that randomisation has been performed will be communicated in an un-blinded fashion to the investigator site staff, and to key members of the central research team, via emails automatically generated by the randomisation website. A researcher (usually the same researcher who conducted the baseline interview) will visit the participant in prison to deliver a letter informing the participant of the randomisation outcome. The researcher will go through the letter with the participant, ensuring that they understand their grouping and when they will be seen next and by whom (researcher or practitioner).

The Intervention

The intervention is designed to engage with individuals with common mental health problems who are close to release, developing a pathway of care in preparation for release and resettlement in the community. The intervention will be delivered in prison between 4 -16 weeks pre-release and for up to 20 weeks post-release. Providing they are still willing to engage, all participants will receive the intervention for eight weeks post-release. However, for those who need further support the intervention can continue for an additional 12 weeks, although at a lower intensity.

Engager is a manualised, person-centred intervention aiming to address mental health needs as well as to support wider issues such as accommodation, education, social relationships and money management. It was developed by bringing together evidence from a realist review,[33] focus groups, case studies and a formative process evaluation. It will be delivered by experienced support workers and supervisor team leaders with experience of therapy. A mentalisation-informed approach underpins all elements of the intervention. Use of existing practitioner skills (e.g. those used in coaching, solution-focused therapy, behavioural activation, Cognitive Behavioural Therapy) is also key to intervention delivery.

At pre-release stage, practitioner and participant will develop a shared understanding of the participant's needs and goals, recognising the links between emotion, thinking, behaviour and social outcomes. A goal attainment plan will be developed and followed, including liaison with relevant agencies and the participant's social networks. Engagement will be maintained throughout the pre-release period; when required, all-day support will be given on release day.

Following release, the practitioner will provide support for the participant to re-enter the community and engage with services. They will continue to work with the participant and any relevant organisations to help them achieve their goals, while encouraging the participant to take responsibility for self-care. The practitioner will also prepare the participant for the end of the intervention, while liaising with relevant community organisations regarding continuity of care.

For peer review only - http://bmjopen.bmj.com/site/about/guidelines.xhtml

Control group

Individuals in the control group will receive care as usual. In prison they will be able to access primary care, mental health and substance misuse services, as would usually occur. They will also receive support from criminal justice and any other third-sector organisations as standard. Their use of health, criminal justice, and third-sector services will be recorded by means of an adapted Client Service Receipt Inventory and medication usage will also be collected from prison medical records and via participant self-report in the community.

Outcome measures

Outcome measure data will be collected at baseline and approximately 1 week pre-release and 1, 3, and 6 months post-release from prison (see Table 2). The primary outcome point is at 6 months post-release. The primary outcome measure is the CORE-OM, a 34-item scale that measures psychological distress. It comprises four domains namely: subjective well-being; depression and anxiety symptoms; general, social and close relationship functioning, and items concerning risk of harm to self or others. Items are rated against how participants felt over the previous week, on a 5-point Likert Scale, with eight items reverse scored.

Secondary outcome measures are as follows:

- Assessment of subjective met/unmet need across key outcome domains using the Camberwell Assessment of Need – Forensic Version (CAN-FOR).[34]
- Change in objective social domains (accommodation, education, employment and benefits).
- Drug and alcohol use using and adapted version of the Treatment Outcomes Profile (TOP).[35]
- Drug and alcohol subjective dependence using the Leeds Dependence Questionnaire (LDQ).[36]
- Service use using an adapted version of the Client Service Receipt Inventory (CSRI).[37]
- Perceived helpfulness of services using the adapted version of the CSRI.
- Generic health related quality of life using the EQ-5D-5L questionnaire.[38]
- Wellbeing related quality of life using the ICEpop CAPability measure for adults (ICECAP-A) questionnaire.[39]
- Experience of care using the Inspire questionnaire (Relationship section only).[40]
- Change in trust, hope and motivation using the Intermediate Outcomes Measurement Instrument (IOMI).[41]
- Proven re-offending rates, based on data from the Police National Computer (PNC).

Due to the nature of the intervention, it will not be possible to blind participants or those delivering the intervention. Attempts to blind researchers during the pilot trial proved challenging, and were largely unsuccessful. Therefore researchers will be aware of which group participants are allocated to, and measures will be implemented to minimise and measure bias, especially for data collection on the primary outcome measure.[29] Specifically, the researchers will use a highly scripted interview for the primary outcome measure, reading each question to the offenders and only deviating from this to clarify the meaning of the question when offenders indicate they do not understand the question.

For peer review only - http://bmjopen.bmj.com/site/about/guidelines.xhtml
For peer review only

Table 2: Tabulated summary of study schedule

		Screening	Baseline	Allocation	Pre-release	Post-release from prison			
**TIMEPOINT**		t_0	t_1		-1 wk t_2	+1 mth t_3	+3 mth[3] t_4	+6 mth t_5	+12 mth t_6
**ENROLMENT:**									
Eligibility screen		X							
Informed consent		X							
PHQ-9		X							
GAD-7		X							
PTSD-Screening Questionnaire		X							
Historical screen for past CMHPs		X							
Allocation[1]				X					
**INTERVENTIONS:**									
Intervention Group:	*Engager Intervention*			◆	—	—	—	◆	
	Usual care	◆	—	—	—	—	—	—	◆
Control Group:	*Usual care*	◆	—	—	—	—	—	—	◆
**ASSESSMENTS:**									
CORE-OM Questionnaire			X			X	X	X	
CORE-10[2]						X	X	X	
CANFOR – Short Version			X				X	X	
Adapted CSRI (including medication)			X		X		X	X	
Objective social outcomes (eg housing)			X					X	
Treatment Outcomes Profile (TOP)			X				X	X	
Leeds Dependence Questionnaire			X					X	
EQ-5D-5L Questionnaire			X				X	X	
ICE-CAP-A Questionnaire			X				X	X	
Intermediate Outcomes Measurement Instrument (IOMI)			X					X	
Standard Assessment of Personality (SAPAS)			X						
Neurodevelopmental Symptoms Rating Scale			X						
Trauma Questionnaire			X						
Contact Sheet			X		X	X			
Brief Inspire Questionnaire					X		X	X	
Police National Computer Offending Data			X						X
**SAFETY MONITORING:**									
Adverse event reporting		◆	—	—	—	—	—	◆	

[1] *Allocation will be performed using a web-based system provided by the CTU, usually within 2 days of completing the screening interview.*

[2] *CORE-10 will only be completed if it is not possible to complete the CORE-OM Questionnaire.*

For peer review only - http://bmjopen.bmj.com/site/about/guidelines.xhtml

Sample size

On the CORE-OM, 5.0 points is the accepted Reliable Change Index in service evaluations.[42,43] In contrast, 2.5 points is held as the upper limit of what would be considered a change compatible with equivalence (Personal communication, Professor Michael Barkham) in trials comparing two interventions. Other trials using the CORE-OM for mental health interventions versus treatment as usual or waiting list controls have achieved mean between group differences in change score of between 3.5 and 7.8.[44,45] A standard deviation (SD) of 5.6 was found in the pilot trial.[29] However, larger clinical studies have reported larger SDs of approximately 7.5.[46]

Given the uncertainty, in both SD and the appropriate minimally clinically important difference (MCID) for the CORE-OM, we calculated sample sizes for different scenarios based on the range of values for these two parameters (see Table 3). This is equivalent to aiming to be able to detect a minimum effect size of 0.26 (i.e. small-to-medium).

Table 3: Sample size (for each group) based on different values of SD and MCID for the CORE-OM*

		Standard deviation (SD)		
		5.5	6.5	7.5
Change to be detected (MCID)	5.0	26	36	48
	4.5	32	44	59
	4.0	40	56	74
	3.5	52	73	97

*At 90% power and 2-sided alpha of 5%

Based on the conservative scenario of a MCID of at least 3.5 and a common SD of 7.5 we will require CORE-OM data on 97 participants in each group at 90% power and 5% alpha. Using an attrition rate of 30%, 140 participants are required per group. Follow-up rates of 63% and 55% were achieved in feasibility and pilot work.[29] However, with learning from the pilot trial, and assistance from the new Community Rehabilitation Companies (CRCs), which now supervise virtually all prison leavers for at least one year, an attrition rate of 30% or less is achievable.

Trial data collection

Trial data will be collected from participants at baseline, 1 week pre-release and at 1, 3 and 6 months post-release. Feasibility and pilot studies highlighted that this population often lead chaotic lives and are difficult to follow-up in the community.[29] To try to address this challenge, the research team will make multiple and sustained attempts to follow-up participants at each time point. Community follow-up interviews will be conducted in a convenient location for the participants, and where appropriate in the premises of services (e.g. National Probation Service or CRC) with which the participant is engaged. Participants will be provided with high street shopping vouchers compensating them for their time at the 3 and 6 month post-release interviews, although this does not apply to participants who have returned to prison and are interviewed there.

The pilot trial highlighted that some participants can be temporarily lost, but subsequently re-emerge (possibly engaging with community services or back in prison).[29] Follow-up data collection points will take place within broad time-windows. The 1 month follow-up will take place between 14 and 60 days post-

For peer review only - http://bmjopen.bmj.com/site/about/guidelines.xhtml

release, the 3 month follow-up will take place between 61 and 151 days post-release, and the 6 month follow-up between 152 and 244 days post-release. Where feasible, follow-ups interviews will take place as close to 1, 3, and 6 months post-release time point as possible. Furthermore, if a participant misses a follow-up interview (e.g. at 3 months), they will continue to be included in the study until all follow-up time-points have lapsed (e.g. 245 days post-release), after which point those remaining out of contact will be regarded as lost. If the research team is in contact with a participant, but setting up a face-to-face interview is challenging (or if they have failed to turn up to an appointment), researchers will attempt to complete the CORE-10[i] by telephone. However, even when the CORE-10 has been completed, researchers will endeavour to follow-up participants with a face-to-face interview.

The numbers and reasons for dropouts and losses to follow-up will be reported for each arm of the study.

Baseline and 1 week pre-release data collection

Baseline data collection will usually continue immediately after the screening interview, although additional sessions can be arranged to meet the needs of individual participants or time constraints within the prison. The following data will be collected:

- Psychological distress using the CORE-OM;
- Subjective rating of need across health and social domains using adapted version of the Camberwell Assessment of Need;
- Healthcare, criminal justice and other service utilisation using an adapted version of the Client Service Receipt Inventory;
- Objective social outcomes (accommodation, education, employment);
- Drug and alcohol use and dependence using the Treatment Outcomes Profile and Leeds Dependence Questionnaire;
- Health related quality of life using the EQ-5D-5L;
- Wellbeing related quality of life using the ICE-CAP-A;
- Intermediate Outcomes Measurement Instrument (IOMI);
- Standardised Assessment of Personality – Abbreviate Scale;
- Neurodevelopmental Symptoms Rating Scale ;
- Experience of traumatic life events using the Trauma Questionnaire.

The questions from the standardised measures, including the primary outcome measure (CORE-OM) will be read out to participants in a precise and consistent manner, to minimise bias and overcome any literacy problems. Questions from the secondary outcome measures are incorporated into a specially constructed flexible interview, which avoids duplication of subject matter in order to reduce disengagement or irritability. Data will be recorded in the Baseline Care Report Form.

In addition to the baseline data collection, the researcher will complete a contact sheet for each participant. This will include contact numbers and addresses provided by the participant, as well as a list of services they are likely to be in contact with post-release. This sheet will be completed in collaboration with the participant, and they will sign the form to consent that the research team can contact them via the relevant services.

The researchers will meet with the participant again within the week prior to their release. The service use table from the adapted Client Service Receipt Inventory, to collect information on services the participant has seen since the baseline data collection and the Brief Inspire Questionnaire will be completed to

For peer review only - http://bmjopen.bmj.com/site/about/guidelines.xhtml
For peer review only

measure the participant's experience of these services. The researcher will also update contact details for the participant.

Information regarding medication prescribed in the 3 months before prison release, and any chronic medical conditions or acute conditions in the previous 12 months, will be collected from the prison healthcare records system. Summary data regarding offence history and number of previous custodial sentences will also be collected from prison records.

Follow-up data collection

At all follow-up meetings, the researcher will remind the participant of the information sheet and consent, drawing attention to data confidentiality and instances of disclosure where the researcher would need to breach confidentiality.

At approximately 1 month post-release, the researcher will contact the participant. This follow-up can be completed by phone, but preferably face-to-face to support continued engagement. The researcher will read aloud to the participant and record responses to the CORE-OM. These data will be used in analysis, but the main objective of the meeting is sustained engagement, and planning further contact.

The 3- and 6-month follow-ups will take place between 61-151 days and 152-244 days post-release respectively, although researchers will endeavour to complete data collection close to the 3 month (90 day) and 6 month (182 day) point. At each time point, participants will be read the questions from the measures listed in Table 2.

Economic Evaluation

The cost-effectiveness of the intervention to increase engagement and access to services, and to improve mental health outcomes will be assessed. This will be compared with service access and support as usual, using the economic model developed in the pilot phase, populated with the trial outcomes and resource use data up to 6-months post-release from prison. It will be conducted from a public sector perspective, initially with the same time horizon as the RCT, and primarily using a cost-consequence approach. Within the cost-consequence approach the estimated incremental costs will be compared with:

- The number of people provided with the service/intervention;
- The incremental differences in the main RCT self-reported health outcomes – CORE scores, and EQ-5D-5L and ICE-CAP social preference weights;
- Incremental differences in the number of ex-prisoners who: have resettled; are in employment; have no proven re-offending; are not homeless;
- Estimated lifetime gains in Quality-Adjusted Life-Years (QALYs) – presuming the persistence of any short-term measured gains and the inclusion of estimated gains associated with social inclusion outcomes such as effective resettlement, increased employment, or reduced re-offending rates.

The cost of providing the intervention will be based on a combination of process of care data collection and intervention practitioner care records and diaries (bottom-up costing approach), and the total costs of service provision (top-down costing). Both deterministic and probabilistic sensitivity analysis will be conducted to explore uncertainty in the model assumptions and parameters, with exploration of key sources of structural uncertainty where feasible.

For peer review only - http://bmjopen.bmj.com/site/about/guidelines.xhtml

The analyses will be conducted according to current guidance from the International Society for Pharmacoeconomics and Outcomes Research (ISPOR) on best practice for conducting trial-based economic evaluation,[47] consistent with the analytical approach used in the statistical analysis of the effectiveness outcomes of the RCT where possible; the cost-effectiveness analysis will be reported in accordance with the Consolidated Health Economic Evaluation Reporting Standards.[48]

Process Evaluation

The Process Evaluation will be conducted in parallel with the trial and will adopt a mixed methods, realist informed, approach.[49] During the development and piloting of the Engager intervention we produced and refined a theoretically informed, and evidence based, logic model of the ways in which the intervention was understood to work,[50] which we intend to test in the process evaluation. The logic model included the core components of the intervention that the practitioners were asked to deliver, the key mechanisms of impact (i.e. how what the practitioners were doing was understood to produce the desired outcomes), and the anticipated outcomes.[51]

Process Evaluation Specific Objectives:

a) To determine the degree to which the core components of the intervention were delivered and the key mechanisms of the intervention occurred;
b) To evaluate the extent to which the core components and key mechanisms of the intervention produced the intended outcomes;
c) To explore any unintended consequences of delivering the intervention;
d) To identify aspects of the intervention and delivery that could be improved;
e) To identify any aspects of intervention delivery that require additional input from practitioner teams when the research team is no longer in place;
f) To develop an understanding of how to deliver the intervention in real world settings (training, supervision, meta-supervision).

Data Collection

The data collection methods were developed, and refined for acceptability, in the pilot trial Formative Process Evaluation and include:

- Semi-structured interviews, with a purposively selected sub-sample of participants, some on one occasion and some at regular intervals throughout their participation in the trial;
- Semi-structured interviews with Engager practitioners and supervisors throughout the trial;
- Semi-structured interviews with other practitioners, and team leaders, in other services about their perceptions of, and interactions with, The Engager practitioners, participants and the intervention;
- Semi-structured interviews with family/partners/friends of participants receiving the Engager intervention;
- Audio-recordings of practitioner group supervision sessions;
- Audio-recordings of selected practitioner-participant interactions;
- Engager practitioner records and notes;
- Quantitative outcome measures, contained within the CRF, and also being used as part of the main trial outcomes
- Ethnographic field notes recorded by the Process Evaluation researchers.

Data Analysis:

The framework analysis methodology, which we developed and applied in the Formative Process Evaluation, will be utilised and extended to collate and interrogate the Process Evaluation data.[52] The deductive components of the framework will be informed by the logic model's key mechanisms of impact; that is the ways in which we understand the intervention to be working. Inductive components of the framework will be surfaces as part of the analytical process. At the end of this analytical process, the logic model of the key mechanisms of impact of the intervention will be revised.

For peer review only - http://bmjopen.bmj.com/site/about/guidelines.xhtml

The Process Evaluation researchers will be distinct from the researchers collecting outcome measures. They will contribute to the qualitative, and therefore more subjective, data collection and the overall analysis. A 'critical friend' researcher, external to the outcome measure and delivery teams, will facilitate the Process Evaluation researchers' opportunity to self-reflexively explore how their presence effects their data collection and experiences in the field which may influence their analytical processes.[51] When the Process Evaluation data and analysis can contribute to refining ongoing fidelity to the Engager model, it will be fed back directly to the intervention delivery team. When the Process Evaluation data and analysis concerns the outcomes of interest, the data will be shared after the trial database has been locked down and initial statistical analyses have been carried out.

If the main trial does not demonstrate that the intervention is effective, additional analysis of the qualitative data will be conducted using thematic methods to explore possible explanations for this,[53] and to glean any additional learning that may have application to other studies with socially marginalised populations and/or those with mental health needs.

Statistical analysis plan

All quantitative data analyses will be conducted and reported in accordance with Consolidated Standards of Reporting Trials (CONSORT) recommendations. We will closely monitor the process of data collection during the trial providing flow diagrams summarising, by group, the numbers approached, recruited, randomised, followed-up/lost to follow up, and outcome completion.

Primary analyses will be conducted on an intention-to-treat basis (i.e. according to randomised group), and compare primary and secondary outcomes at 6-month follow up between randomised groups on those with complete data sets. Outcomes will be compared using linear regression based methods, adjusting for baseline outcome scores and stratification variables. Where necessary, outcomes will be transformed to ensure good regression model fit. A secondary analysis will compare primary and secondary outcomes between groups at all follow-up time points using a repeated measures approach. Reasons for missing data (including loss to follow-up and participant drop-out) will be documented, and the baseline characteristics of those with and without missing data compared. Using different assumptions for missing data, we will undertake sensitivity analyses using various imputation models, comparing between group results to the completers primary analysis. We shall also explore the possibility of conducting secondary per protocol between group comparisons. If possible this will be based on a pre-defined minimum level of intervention receivership, and using Complier Average Causal Effect analysis methods. The analyst will be blinded to group allocation and the analysis will be undertaken using STATA v.14.2.

A detailed statistical analysis plan will be prepared before any data analysis is conducted. The statistical analysis plan will be agreed with the Trial Steering Committee.

Trial management and independent committees

Members of the research team directly involved with the day-to-day running of the trial will meet fortnightly to discuss trial progress, with additional email and telephone exchanges as required. A full Trial Management Group including health economists, statisticians, process evaluation researchers, and a sponsor representative will meet quarterly to review trial progress.

The Engager Trial Steering Committee (Chair: Professor Pamela Taylor, and three other independent members including a patient and public involvement representative) will meet 1-2 times per year to

For peer review only - http://bmjopen.bmj.com/site/about/guidelines.xhtml

oversee the conduct of the trial, safety and ethics. The Trial Steering Committee formally agreed that given the social/psychological nature of the intervention, only limited safety monitoring would be required, and therefore an independent Data Monitoring Committee was not required.

Ethics and Dissemination

We have obtained ethical approval (Wales REC 3, reference: 15/WA/0314), National Offender Management Service (NOMS; ref: 2015-283) approval and local Trust governance approvals for each site (Devon Partnership NHS Trust, Dorset Hospital University Foundation NHS Trust and Lancashire Care NHS Foundation Trust). The study has also been adopted by the NIHR Clinical Research Network.

The trial will be conducted in accordance with the ethical principles outlined in the Declaration of Helsinki, and those consistent with GCP. The Trial Steering Committee will ensure adherence to these guidelines. Any amendments to the protocol will be submitted for ethical approval as appropriate.

Findings will be published in peer-reviewed journals and presented at local, national, and international conferences to publicise and explain the research to key audiences. A final report will be submitted to the National Institute for Health Research.

References

1. C. Brooker, J. Repper, C. Beverley, M. Ferriter, N. Brewer. *Mental health services and prisoners: A review*, School of Health and Related Research (ScHARR), University of Sheffield, Sheffield, 2002.
2. D. Grubin, L. Birmingham, D. Mason. *The Durham Remand Study,* University of Newcastle and Newcastle City Health Trust, Her Majesty's Prison Service/ Northern and Yorkshire Regional Health Authority, London, 1997.
3. M. Rotter, B. Way, M. Steinbacher, D. Sawyer, H. Smith. *Personality Disorders in Prison: Aren't They All Antisocial?*, Psychiatr Q, 2002, 73, 1573-6709.
4. S. Fazel and J. Danesh, *Serious mental disorder in 23 000 prisoners: a systematic review of 62 surveys,* Lancet, 2002, 359:545-550.
5. N. Singleton, H. Meltzer, R. Gatward, J. Coid, D. Deasy. *Psychiatric morbidity among prisoners in England and Wales*, The Stationery Office, London, 1998.
6. D. Pratt, M. Piper, L. Appleby, R. Webb, J. Shaw. *Suicide in recently released prisoners: a population-based cohort study*, Lancet, 2006, 368:119-123.
7. A. Howerton, R. Byng, J. Campbell, D. Hess, C. Owens, P. Aitken. *Understanding help seeking behaviour among male offenders: qualitative interview study*, BMJ, 2007, 334, 267-268.
8. R. Byng, C. Weyer Brown, R. Sheaff, C. Samele, S. Duggan, D. Harrison, C. Owens, P. Smithson, C. Wright, J. Annison, C. Brown, R. Taylor, W. Henley, A. Qureshi, D. Shenton, I. Porter, C. Warrington, J. Campbell. *Care for Offenders: Continuity of Access*, NIHR Service Delivery and Organisation Programme SDO Project 08/1713/210, 2012.
9. R. Walmsley, L. Howard, S. White. *The National Prison Survey 1991: Main Findings*. Home Office Research Study 128, HMSO, London, 1992.
10. S. Niven, J. Olagundoye, *Jobs and homes: A survey of prisoners nearing release.* Home Office Research Findings 173, Home Office, London, 2002.
11. S. Niven, D. Stewart, *Resettlement outcomes on release from prison in 2003.* Home Office Research Findings 248, Home Office, London, 2005.
12. Social Exclusion Unit. *Reducing re-offending by ex-prisoners: Report by the Social Exclusion Unit*, Social Exclusion Unit, London, 2002.
13. D. Stewart. *The problems and needs of newly sentenced prisoners: Results from a national survey*, Ministry of Justice, London, 2008.

For peer review only - http://bmjopen.bmj.com/site/about/guidelines.xhtml

14. Ministry of Justice. *Proven re-offending statistics quarterly, April 2014 to March 2015*. Ministry of Justice, 2017.
15. Home Office. *IOM Efficiency Toolkit Phase 2: revised unit costs of crime and multipliers*, Home Office, 2011.
16. J. Steel, G. Thornicroft, L. Birmingham, C. Brooker, A. Mills, M. Harty, J. Shaw. *'Prison Mental Health Inreach Services'*, British Journal of Psychiatry, 2007, 190:373-374.
17. J. Shaw, *Management of released prisoners with severe and enduring mental illness: Adaptation of the Critical Time Intervention*, 2005.
18. M. Williamson. *Improving the health and social outcomes of people recently released from prisons in the UK: A perspective from primary care*, The Sainsbury Centre for Mental Health, London, 2006.
19. J. Hague, A. Cohen. *The neglected majority: Developing intermediate mental health care in primary care*, London, 1995.
20. Department of Health. *Improving access to psychological therapies: Offenders - positive practice guide*, Department of Health, London, 2009.
21. NICE. *Antisocial personality disorder: Treatment, management and prevention*, National Institute for Health and Clinical Excellence, London, 2009.
22. NICE. *Borderline personality disorder: Treatment and management*, National Institute for Health and Clinical Excellence, London, 2009.
23. G. Durcan. Criminal Justice and Mental Health: Experiences across England and Wales, Centre for Mental Health, London, 2016.
24. W. Vanderplasschen, J. Wolf, C. G. Rapp, E. Broekaert. *Effectiveness of different models of case management for substance-abusing populations,* Journal of Psychoactive Drugs, 2007, 39:81-95.
25. E. Harvey, A. Shakeshaft, K. Hetherington, C. Sannibale, R. P. Mattick. *The efficacy of diversion and aftercare strategies for adult drug-involved offenders: a summary and methodological review of the outcome literature,* Drug Alcohol Review, 2007, 26:379-387.
26. S. Lewis, M. Maguire, P. Raynor, M. Vanstone, J. Vennard. *'What Works in Resettlement? Findings from Seven Pathfinders for Short-term Prisoners in England and Wales'*, Criminology and Criminal Justice, 2007, 7:33-53.
27. M. Maguire, K. Holloway, M. Liddle, F. Gordon, P. Gray, A. G. Smith, S. Wright, *Evaluation of the Transitional Support Scheme*, Welsh Assembly Government, Cardiff, 2010.
28. C. Evans, J. Mellor-Clark, F. Margison, M. Barkham, K. Audin, J. Connell, G. McGrath. *CORE: Clinical Outcomes in Routine Evaluation*, Journal of Mental Health, 2000, 9(3):247-255.
29. C. Lennox, T. Kirkpatrick, R. S. Taylor, R. Todd, C. Greenwood, M. Haddad, C. Stevenson, A. Stewart, D. Shenton, L. Carroll, S. L. Brand, C. Quinn, R. Anderson, M. Maquire, T. Harris, J. Shaw, R. Byng. *Pilot Randomised Controlled Trial of the ENGAGER collaborative care intervention for prisoners with common mental health problems, near to and after release*, Pilot and Feasibility Studies, 2017 [submitted].
30. K. Kroenke, R. L. Spitzer, J. B. Williams. *The PHQ-9: validity of a brief depression severity measure*, Journal of General Internal Medicine, 2001, 16:606-613.
31. R. L. Spitzer, K. Kroenke, J. B. W. Williams, B. Lowe, *A brief measure for assessing Generalised Anxiety Disorder: The GAD-7,* Archives of Internal Medicine, 2006, 166:1092-1097.
32. A. Prins, P. Ouimette, R. Kimerling, R.P. Cameron, D.S. Hugelshofer, J. Shaw-Hegwer, A. Thrailkill, F.D. Gusman, J.I. Sheikh. *The primary care PTSD screen (PC-PTSD): Development and operating characteristics,* Primary Care Psychiatry, 2003, 9:9-14.
33. M. Pearson, S. L. Brand, C. Quinn, J. Shaw, M. Maguire, S. Michie, S. Briscoe, C. Lennox, A. Stirzaker, T. Kirkpatrick, R. Byng. *Using realist review to inform intervention development: methodological illustration and conceptual platform for collaborative care in offender mental health*, Implementation Science, 2015, 28(10):134.
34. S. Thomas, M.A. Harty, J. Parrott, P. McCrone, M. Slade, G. Thornicroft, *CANFOR: Camberwell Assessment of Need-Forensic Version*, Royal College of Psychiatrists, London, Gaskell, 2003.
35. J. Marsden, M. Farrell, C. Bradbury, A. Dale-Perera, B. Eastwood, M. Roxburgh, S. Taylor, *Development of the treatment outcomes profile*, Addiction, 2008, 103:1450-60.

For peer review only - http://bmjopen.bmj.com/site/about/guidelines.xhtml

36. D. Raistrick, J. Bradshaw, G. Tober, J. Weiner, J. Allison, C. Healey. *Development of the Leeds Dependence Questionnaire (LDQ): a questionnaire to measure alcohol and opiate dependence in the context of a treatment evaluation package,* Addiction, 1994, 89(5):563-72.
37. J. Beecham, M. Knapp. *Costing Psychiatric Interventions*, *in Measuring Mental Health Needs*, G. Thornicroft, C. Brewin, J. Wing (Eds.), Gaskell, 1992.
38. EuroQoL Group. *EuroQoL: a new facility for the measurement of health-related quality of life*, Health Policy, 1990, 16:199–208.
39. H. Al-Janabi, T. Flynn, J. Coast. *Development of a self-report measure of capability wellbeing for adults: the ICECAP-A,* Quality of Life Research, 2012, 21(1):167-176.
40. J. Williams, M. Leamy, V. Bird, C. Le Boutillier, S. Norton, F. Pesola, M. Slade. *Development and evaluation of the INSPIRE measure of staff support for personal recovery*, Social Psychiatry Psychiatriatric Epidemiol, 2015, 50(5):777-86.
41. M. Maguire, E. Disley, M. Liddle, R. Meek, N. Burrowes, G. Lewis. *Developing a toolkit to measure intermediate outcomes to reduce reoffending,* London: NOMS, 2017, Ministry of Justice Analytical Series.
42. N.S. Jacobson, P. Truax. *Clinical significance: A statistical approach to defining meaningful change in psychotherapy research*, Journal Consultant Clinical Psychology, 1991, 59(1):12–19.
43. J. Connell, M. Barkham, W.B. Stiles, E. Twigg, N. Singleton, O. Evans, J. N. V. Miles. *Distribution of CORE-OM scores in a general population, clinical cut-off points and comparison with the CIS-R,* British Journal of Psychiatry, 2007, 190,:69–74.
44. C. Thiels, U. Schmidt, J. Treasure, R. Garth, N. Troop. *Guided self-change for bulimia nervosa incorporating use of a self-care manual,* American Journal of Psychiatry, 1998, 155:947 -953.
45. L.B. Nordgren, E. Hedman, J. Etienne, J. Bodin, A. Kadowaki, S. Eriksson, E. Lindkvist, G. Andersson, P. Carlbring. *Effectiveness and cost-effectiveness of individually tailored Internet-delivered cognitive behavior therapy for anxiety disorders in a primary care population: a randomized controlled trial,* Behavioural Research and Therapy, 2014, 59:1–11.
46. M. Lucock, K. Padgett, R. Noble, A. Westley, C. Atha, C. Horsefield, C. Leach. *Controlled Clinical Trial of a Self-Help for Anxiety Intervention for Patients Waiting for Psychological Therapy*, *Behavioural and Cognitive Psychotherapy* , 2008, 36(5):541-551.
47. S.D. Ramsey, R.J. Willke, H.A. Glick, S.D. Reed, F. Augustovski, B. Jonsson, A. Briggs, S.D. Sullivan. *Cost-effectiveness analysis alongside clinical trials II: An ISPOR good research practices task force report*. Value in Health, 2015, 18:161-172.
48. D. Husereau, M. Drummond, S. Petrou, C. Carswell, D. Moher, D. Greenberg, F. Augustovski, A.H. Briggs, J. Mauskopf, E. Loder. *Consolidated Health Economic Evaluation Reporting Standards (CHEERS) statement.* BMJ, 2013; 346:f1049.
49. R. Pawson and N. Tilley. *Realistic Evaluation*, London, Sage Publications, 1997.
50. G. Moore, S. Audrey, M. Barker, L. Bond, C. Bonnell, W. Hardeman, L. Moore, A. O'Cathain, T. Tinati, D. Wight, J. Baird. *Process evaluation of complex interventions: Medical Research Council guidance*, MRC Population Health Science Research Network, London, 2014.
51. P. Craig, P. Dieppe, S. Macintyre, S. Michie, I. Nazareth, M. Petticrew. Developing and evaluating complex interventions: the new Medical Research Council guidance, BMJ, 2008, 337:1655.
52. J. Ritchie, L. Spencer. *"Qualitative data analysis for applied policy research"* in A. Bryman and R.G. Burgess (Eds.), Analysing Qualitative Data, London, Routledge, 1994, 173-94.
53. V. Braun, V. Clarke. *"Using thematic analysis in psychology"*, Qualitative Research in Psychology, 2006, 3(2):77-101.

Author's contribution

TK, CS and LC wrote the first draft of the manuscript. RB is the Chief Investigator on the NIHR grant and RST, RA, MM, MH, SM, CO, GD, AS, WH, CQ, TH, MP and JS are co-applicants on the grant and contributed to the conceptualisation of the study design. CL, SLB, ASte, RT, SR-B, and RG are members of the study

For peer review only - http://bmjopen.bmj.com/site/about/guidelines.xhtml

team that have contributed to the analysis of pilot data and development of the trial methodology. All authors provided critical evaluation of the manuscript and have given final approval of the manuscript.

Funding statement

Engager is funded by the National Institute for Health Research (NIHR) under its Programme Grant for Applied Research Programme (Grant number: RP-PG-1210-12011). This research was also supported by the National Institute for Health Research (NIHR) Collaboration for Leadership in Applied Health Research and Care South West Peninsula at the Royal Devon and Exeter NHS Foundation Trust. The views expressed are those of the author(s) and not necessarily those of the NHS, the NIHR or the Department of Health. The funder had no role in the design of this study and will not have any role during execution, analysis, interpretation of findings, or decision to submit results. The views expressed are those of the authors and not necessarily those of the NHS, the NIHR, or the Department of Health.

Competing Interests

The authors declare that they have no competing interests.

[i] The CORE-10 will be used when completing the measure over the phone because it is shorter. The 34-item CORE-OM was considered too long to complete over the phone with this population of participants.

For peer review only

BMJ Open

Evaluation of a complex intervention (Engager) for prisoners with common mental health problems, near to and after release – study protocol for a randomised controlled trial.

Journal:	*BMJ Open*
Manuscript ID	bmjopen-2017-017931.R1
Article Type:	Protocol
Date Submitted by the Author:	29-Aug-2017

Complete List of Authors:	Kirkpatrick, Tim; Plymouth University, Peninsula Schools of Medicine and Dentistry<br>Lennox, Charlotte; University of Manchester, Manchester Academic Health Science Centre<br>Taylor, Rod; University of Exeter, Exeter Medical School<br>Anderson, Rob; University of Exeter, Peninsula Technology Assessment Group (PenTAG)<br>Maguire, Michael; University of South Wales, Centre for Criminology<br>Haddad, Mark; Centre for Mental Health Research, Professor of Criminology<br>Michie, Susan; University College London, Centre for Outcomes Research and Effectivenes<br>Owens, Christabel ; University of Exeter Medical School , Institute of Health Research<br>Durcan, Graham; Centre for Mental Health<br>Stirzaker, Alex; Avon and Wiltshire Mental Health Partnership NHS Trust, Associate Director for Criminal Justice Programme<br>Henley, W; Institute of Health Research, University of Exeter Medical School, College House, St Luke's Campus, Exeter, Devon, UK, EX1 2LU<br>Stevenson, Caroline; The University of Manchester<br>Carroll, Lauren; Plymouth University Peninsula Schools of Medicine and Dentistry<br>Quinn, Cath; Plymouth University Peninsula Schools of Medicine and Dentistry, Primary Care, Clinical Trials and Population Studies<br>Brand, Sarah; Plymouth University Peninsula Schools of Medicine and Dentistry<br>Harris, Tirril; Kings College, University of London, Socio-Medical Research Group, Institute of Psychiatry<br>Stewart, Amy; Plymouth University Peninsula Schools of Medicine and Dentistry, Primary Care, Clinical Trials and Population Studies<br>Todd, Roxanne; The University of Manchester, Division of Psychology & Mental Health<br>Rybczynska-Bunt, Sarah; Plymouth University Peninsula Schools of Medicine and Dentistry, Primary Care, Clinical Trials and Population Studies<br>Greer, Rebecca; Plymouth University Peninsula Schools of Medicine and Dentistry, Primary Care, Clinical Trials and Population Studies<br>Pearson, Mark; University of Exeter Medical School, University of Exeter, Peninsula Technology Assessment Group (PenTAG)<br>Shaw, Jenny; University of Manchester, Academic Health Science Centre<br>Byng, Richard; Plymouth University Peninsula Schools of Medicine and

	Dentistry, Primary Care, Clinical Trials and Population Studies
**Primary Subject Heading**:	Mental health
Secondary Subject Heading:	Health services research
Keywords:	Prison, Randomised controlled trial, MENTAL HEALTH, Resettlement, Protocol

For peer review only

Evaluation of a complex intervention (Engager) for prisoners with common mental health problems, near to and after release – study protocol for a randomised controlled trial.

T Kirkpatrick, C Lennox, RS Taylor, R Anderson, M Maguire, M Haddad, S Michie, C Owens, G Durcan, A Stirzaker, W Henley, C Stevenson, L Carroll, C Quinn, SL Brand, T Harris, A Stewart, R Todd, S Rybczynska-Bunt, R Greer, M Pearson, J Shaw, R Byng

Corresponding author:
Dr T Kirkpatrick:
Plymouth University Peninsula Schools of Medicine and Dentistry,
N9 Plymouth Science Park,
Davy Road,
Derriford,
Plymouth,
PL6 8BX
Email: tim.kirkpatrick@plymouth.ac.uk
Phone: 01752 764477
Fax: 01752 764234

Co-authors:
Division of Psychology and Mental Health, 2.315 Jean McFarlane Building, The University of Manchester, Oxford Road, Manchester, M13 9PL C Lennox, Research Fellow and Lecturer University of Exeter Medical School, St Luke's Campus, Heavitree Road, Exeter EX1 2LU RS Taylor Chair of Health Services Research Exeter Medical School, St Luke's Campus, Heavitree Road, Exeter EX1 2LU R Anderson Associate Professor Centre for Criminology, University of South Wales, Pontypridd, CF37 1DL M Maguire Professor of Criminology Centre for Mental Health Research, School of Health Sciences, City, University of London, Northampton Square, London EC1V 0HB M Haddad Senior Lecturer UCL Centre for Behaviour Change, University College London, 1-19 Torrington Place, London WC1E 7HB S Michie Director of UCL Centre for Behaviour Change University of Exeter Medical School, St Luke's Campus, Heavitree Road, Exeter EX1 2LU C Owens Associate Professor Centre for Mental Health, Unit 2D21 Technopark, LSBU 90 London Road, London SE1 6LN G Durcan Associate Director for Criminal Justice Program Avon and Wiltshire Mental Health Partnership NHS Trust, LIFT Psychology, Old Town Surgery, Curie Avenue, Swindon SN1 4GB A Strizaker Clinical Psychologist University of Exeter Medical School, St Luke's Campus, Heavitree Road, Exeter EX1 2LU W Henley Professor of Medical Statistics Division of Psychology and Mental Health, 2.315 Jean McFarlane Building, The University of Manchester, Oxford Road, Manchester, M13 9PL C Stevenson Research Assistant Plymouth University Peninsula Schools of Medicine and Dentistry, N9 Plymouth Science Park, Davy Road, Derriford, Plymouth, PL6 8BX L Carroll Research Assistant Plymouth University Peninsula Schools of Medicine and Dentistry, N9 Plymouth Science Park, Davy Road, Derriford, Plymouth, PL6 8BX C Quinn Senior Research Fellow Plymouth University Peninsula Schools of Medicine and Dentistry, N9 Plymouth Science Park, Davy Road, Derriford, Plymouth, PL6 8BX SL Brand Research Fellow Socio-medical Research Group, Institute of

For peer review only - http://bmjopen.bmj.com/site/about/guidelines.xhtml
For peer review only

Psychiatry, Kings College, University of London T Harris Honorary Research Fellow Plymouth University Peninsula Schools of Medicine and Dentistry, N9 Plymouth Science Park, Davy Road, Derriford, Plymouth, PL6 8BX A Stewart Research Assistant Division of Psychology and Mental Health, 2.315 Jean McFarlane Building, The University of Manchester, Oxford Road, Manchester, M13 9PL R Todd Research Assistant Plymouth University Peninsula Schools of Medicine and Dentistry, N9 Plymouth Science Park, Davy Road, Derriford, Plymouth, PL6 8BX S Rybczynska-Bunt Research Fellow Plymouth University Peninsula Schools of Medicine and Dentistry, N9 Plymouth Science Park, Davy Road, Derriford, Plymouth, PL6 8BX R Greer Research Assistant University of Exeter Medical School, St Luke's Campus, Heavitree Road, Exeter EX1 2LU M Pearson Senior Research Fellow Division of Psychology and Mental Health, 2.315 Jean McFarlane Building, The University of Manchester, Oxford Road, Manchester, M13 9PL J Shaw Professor of Forensic Psychiatry Plymouth University Peninsula Schools of Medicine and Dentistry, N9 Plymouth Science Park, Davy Road, Derriford, Plymouth, PL6 8BX R Byng Professor of Primary Care Research

Key words

Prison, resettlement, Randomised controlled trial, mental health, protocol

Word count: 6440 (+284 in abstract)

For peer review only

ABSTRACT

Introduction

The ‘Engager’ programme is a ‘through-the-gate’ intervention designed to support prisoners with common mental health problems as they transition from prison back into the community. The trial will evaluate the clinical and cost effectiveness of the Engager intervention.

Methods and Analysis

The study is a parallel two-group randomised controlled trial (RCT) with 1:1 individual allocation to either: a) the Engager intervention plus standard care (intervention group), or b) standard care alone (control group) across two investigation centres (South West and North West of England). Two hundred and eighty prisoners meeting eligibility criteria will take part. Engager is a person-centred complex intervention delivered by practitioners and aimed at addressing offenders’ mental health and social care needs. It comprises one-to-one support for participants prior to release from prison and for up to 20 weeks post-release. The primary outcome is change in psychological distress measured by the Clinical Outcomes in Routine Evaluation – Outcome Measure (CORE-OM) at six months post-release. Secondary outcomes include: assessment of subjective met/unmet need, drug and alcohol use, health related quality of life, and wellbeing related quality of life measured at three and six months post-release; change in objective social domains, drug and alcohol dependence, service utilisation, perceived helpfulness and change in trust, hope and motivation at six months post-release; and recidivism at 12 months post release. A process evaluation will assess fidelity of intervention delivery, test hypothesised mechanisms of action and look for unintended consequences. An economic evaluation will estimate the cost-effectiveness.

Ethics and Dissemination

This study has been approved by the Wales Research Ethics Committee 3 (ref: 15/WA/0314) and the National Offender Management Service (NOMS; ref: 2015-283). Findings will be disseminated to commissioners, clinicians and service users via papers and presentations.

Trial registration: ISRCTN11707331. Registration date 04/02/2016

[284 words, excluding trial registration details]

For peer review only

Strengths and limitations of this study

- The study will be a two-centre, randomised controlled trial of a through-the-gate intervention for prisoners with common mental health problems; it will provide much needed evidence of what works for this difficult to engage population.
- The primary and secondary outcomes have been selected following extensive piloting work and cover a broad range of outcome domains that could be impacted by the complex intervention.
- The study adopts a flexible and pragmatic approach to data collection to try to overcome the challenges of following up this population after release from prison.
- The lack of blinding of researchers collecting study data is a limitation of the study design.

For peer review only - http://bmjopen.bmj.com/site/about/guidelines.xhtml

INTRODUCTION

This paper presents the protocol for a randomised control trial (RCT) to test the effectiveness of a complex 'through the gate' intervention for prisoners with common mental health problems. RCTs in prison settings are rare,[1] and we are unaware of any which have focused on responses to common mental health problems. This is a surprising omission, given that the point prevalence of mental health problems among prison populations has been reported as between 50 and 90% both in the UK,[2-4] and internationally.[5] In England and Wales, an Office of National Statistics survey reported high rates of personality disorder (PD) (64%), neurotic disorders (40%), drug dependency (43%) and hazardous alcohol use (63%) in sentenced prisoners, with higher rates generally being found in remand prisoners.[6] High levels of suicide, suicidal thoughts and self-harming behaviour have also been reported among both prisoners and ex-prisoners[7], with the risk of suicide for male offenders leaving prison being eight times the national average.[8,9] Our development work indicated high rates of anxiety and depression, with 47% reaching likely caseness for anxiety, PTSD or depression while in prison, of which 32% were still 'cases' after release. There was also substantial co-morbidity, especially with substance abuse.[10]

In addition to mental health problems, offenders have wide-ranging personal and social problems, including homelessness, unemployment, broken relationships with both partners and children, and they typically live chaotic lives. In our previous cross-sectional study of 200 offenders (100 serving prison sentences and 100 serving community sentences), 37% reported problems with family relationships; the majority of the sample were unemployed or on long-term sickness benefit (65% in prison and 70% in the community sample), and 26% had on-going legal or criminal justice issues.[11] These results echo previous surveys of prisoners.[12-15] The Surveying Prisoner Crime Reduction (SPCR) study, which is based on regular interviews with a cohort of prisoners in England and Wales before and after release, shows two-thirds reporting unemployed status before going into custody, and 37% reporting the need for assistance in finding accommodation on release.[16] These issues tend to be the focus of offenders' own concerns, indicating a crucial need to address them, as well as providing motivation for change. The international literature identifies similar constellations of inter-related personal and social problems among numerous prisoners leaving custody, and similar challenges facing resettlement (or 'reentry') services.[17-21]

The cost of failing to address these issues is high. Those serving short-term sentences place a considerable burden on society. Twelve-month proven re-offending rates for short-sentence prisoners are currently close to 60%[22] and, in addition to the distress and inconvenience commonly experienced by their victims, many 'volume' offences have a surprisingly high financial impact. For example, in 2010 the costs of an average domestic burglary were estimated at £3,925, and of a less serious wounding at £9,790.[23] Therefore, the potential benefits of addressing these issues, to individuals and communities, as well the financial savings, are significant.

There are complex relationships between mental health, substance misuse, social exclusion and criminal behaviour. However, these tend to be studied separately, with interventions designed to address them being developed and delivered in isolation. An underpinning aim of the Engager intervention is to identify and overcome service barriers, particularly between health and criminal justice sectors, and embed multi-agency working within the intervention.

In the UK, prison healthcare is often provided by a number of different NHS, private, or third sector organisations, each providing separate primary care, mental health care, drug and alcohol services. Opiate substitution services are now generally available in prison, and mechanisms for achieving continuity post-

For peer review only - http://bmjopen.bmj.com/site/about/guidelines.xhtml

release are improving. Mental health services for those with severe and enduring mental illness have faced considerable challenges,[24] but have improved care for those with psychosis, with new evidence now supporting the development of mental health pathways on release.[25]

By contrast, provision of psychological therapy for offenders with common mental health problems is limited in both prison and community settings.[11] The prison environment complicates diagnostic assessment,[4] and for some fewer stressors in prison may reduce anxiety, making it difficult to identify mental health problems that may arise post-release. The focus of hard-pressed prison healthcare staff is on immediate concerns, rather than longer-term post-release planning. Improving Access to Psychological Therapies (IAPT) services in prisons are still in the early stages of development.

Once released into the community, ex-prisoners with common mental health problems are, in theory, provided for by mainstream statutory services including general practice, community mental health teams and IAPT services. In reality, few access these services. For example, in a previous study we found an average of only 0.96 contacts with mental health services per offender per year for those reporting common mental health problems,[11] suggesting that a lack of care on release is the norm.[26,27]

Despite negligible uptake and high need, no systems worldwide have been identified for actively engaging offenders with common mental health problems whilst in prison, providing initial treatment and transferring care to community teams. Many ex-prisoners, like others with common mental health problems complicated by co-morbidity, fall between primary care, IAPT and specialist services[28-31]. They are further disadvantaged by their reluctance both to seek help and to accept mental health diagnoses, and by lower levels of GP registration. [10,11,26] Services can also be seen as resistant to offenders, and are not designed to meet the needs of those with complex and multiple vulnerabilities.[32] This contrasts with well-established services, together with arrangements for transfer of care, for those with opiate misuse. [33,34] In a relatively small proportion of cases, psychological input and/or general support is provided by statutory or third-sector resettlement services, from probation-delivered thinking skills 'booster' programmes for prisoners on licence, through to volunteer or peer-mentoring services, [35,36] although currently resettlement plans typically contain limited reference to health concerns.

In view of these factors, provision of care for common mental health problems should be considered as part of the range of services making up collaborative care and directed towards improving social outcomes and resettlement. The Engager research programme was designed to develop and evaluate a collaborative care intervention for prisoners with common mental health problems, near to and after release from prison, supporting multiple needs rather than focused on specific diagnoses or on a particular therapy. We describe the methods of the Engager trial here.

AIMS AND HYPOTHESIS

The Engager trial aims to answer the research question: *What is the effectiveness and cost-effectiveness of the Engager Intervention plus usual care, compared to usual care alone, in prisoners with common mental health problems, both before release and for between 3 and 5 months following release from prison?* The primary hypothesis is that the participants receiving the Engager intervention plus usual care (the 'intervention group') will have reduced levels of psychological distress as measured by the Clinical Outcomes in Routine Evaluation – Outcome Measure (CORE-OM)[37] at six months post-release from prison (primary outcome) compared to participants receiving usual care alone (the 'control group').

For peer review only - http://bmjopen.bmj.com/site/about/guidelines.xhtml

Secondary hypotheses of the trial are that, compared to the control group, the intervention group will have:

- an increase in the number of subjective met needs and decrease in the number of unmet needs in relation to accommodation, education, work/money/benefits, family/friends/company/intimacy, physical and mental health, safety to self, and self-care, safety to others, and leisure activities;
- a decrease in substance use and subjective view of dependence;
- improvement in generic health related quality of life, wellbeing related quality of life, subjective experience of care received and perceived helpfulness of services;
- a reduction in levels of proven re-offending.

The trial will estimate the total cost, and per prisoner cost, of providing the Engager intervention, and the cost-effectiveness of the Engager intervention plus usual care versus usual care alone across health, social care, and criminal justice sectors.

The trial also includes a parallel process evaluation, designed to: determine the degree to which the core mechanisms of the intervention were delivered; evaluate the extent to which the core mechanisms of the intervention produced the intended outcomes; identify aspects of the intervention and delivery that could be improved, and explore unintended consequences of the intervention.

METHODS AND ANALYSIS

Design

The study is a parallel two-group randomised controlled trial (RCT) with 1:1 individual participant allocation to either the Engager Intervention plus standard care (intervention group) or standard care alone (control group), with economic evaluation and parallel process evaluation. The trial is registered as ISRCTN11707331 (04/02/2016).

Setting

The study will be conducted in two investigation centres (South West and North West of England). Participants will be recruited from three prisons, two in the South West and one in the North West of England. Participants will be recruited in equal numbers from each of the two investigation centres, for both the intervention and control groups. Recruitment and baseline interviews will take place in the prisons, with follow-up interviews taking place in a suitable community location or (for those who are back in prison) in prison. Conduct of the trial in each centre will be led by a local Principal Investigator, supported by a research team, all of whom have received training in Good Clinical Practice and the requirements of the study protocol.

Study population

Potential participants will be men serving a custodial sentence of two years or less, who are within 4–20 weeks from release from prison and who are being released to the geographical area of the study. Potential participants will be identified using the Prison National Offender Management Information System (PNOMIS). Female prisoners, men on remand, and those with a diagnosis of serious mental illness

For peer review only - http://bmjopen.bmj.com/site/about/guidelines.xhtml

or on the Offender Personality Disorder Pathway will be excluded from the trial. The full list of participant inclusion and exclusion criteria is provided in Table 1.

Table 1 – Trial entry criteria

Inclusion criteria
• Men with prison sentences of up to and including two years.<br>• Being released to the geographical area of the study.<br>• Having between 4 and 20 weeks remaining to serve in prison.<br>• Willing to engage with treatment services and research procedures.<br>• Identified using screening instruments as having, or likely to have following release, common mental health problems.
**Exclusion criteria**
• Men on remand.<br>• Women (numbers are smaller, and prisons are remote; resettlement needs are different; research procedures developed are not feasible for this context). Research will be in male prisons only.<br>• Those with serious and enduring mental disorder and/or on the caseload of the prison in-reach team.<br>• Those with active suicidal intent requiring management under the safer custody process or prison in-reach team, and where the healthcare team managing the prisoner feels it would be detrimental. Once risk levels reduce individuals in this group will be eligible if not excluded for another reason.<br>• Those with primary personality disorder who are on the caseload of the Offender Personality Disorder Pathway programme.<br>• Those who present a serious risk of harm to the researchers or intervention practitioners.<br>• Those unable to provide informed consent.

Individuals will be approached up to 20 weeks prior to release. Initial contact will be made by a member of the research team. They will be given the Participant Information Sheet (see supplementary file – Appendix 1 - Information sheet for RCT V5 14.03.17)and the researcher will discuss any queries/concerns with them. Researchers will take consent (see supplementary file - Appendix 2 - Consent form for RCT V5 24.03.2017) from individuals who wish to participate. All individuals providing written informed consent will complete a short screening interview to identify those currently experiencing common mental health problems, or who have experienced common mental health problems in the previous two years that impacted on their day-to-day functioning, and are likely to experience similar problems on release. The screening interview comprises the Patient Health Questionnaire-9 (PHQ-9),[38] the Generalised Anxiety Disorder-7 (GAD-7),[39] the Primary Care PTSD Screen (PC-PTSD),[40] and a bespoke Historical Common Mental Health Problem screen. The PHQ-9, GAD-7 and PC-PTSD are routinely used in IAPT services, are quick and easy to administer screening tools for depression, anxiety and PTSD respectively. The researcher will read the questions to the participants, using a narrative conversational format developed in our pilot work to facilitate engagement.[41] Individuals will be considered suitable for inclusion in the study if the screening interview indicates that they:

- Have a current common mental health problem as indicated by a score of 10 or more on the PHQ-9 or GAD-7, or 3 or more on the PC-PTSD;

or

For peer review only - http://bmjopen.bmj.com/site/about/guidelines.xhtml

- Have experienced a common mental health problem during the past two years which prevented them from functioning normally in everyday tasks, and which is likely to be a problem for them again following their release[i].

If a participant screens in following this assessment they will continue to the full baseline interview. Participants will be informed that participation is voluntary and that they are free to withdraw from the study at any time, and it is stressed that withdrawal from the study will not affect their legal rights. They will also be informed that the researcher has a duty to inform prison staff if they disclose certain information, such as intent to harm self or others.

Randomisation

Participants will be individually randomised in a 1:1 ratio to receive either the Engager Intervention in addition to usual care, or usual care alone. Randomisation will be achieved by means of a web-based system created by Peninsula Clinical Trials Unit (PenCTU). Randomisation numbers will be computer-generated and assigned in strict sequence. Randomisation will be stratified to ensure balance between the two treatment arms across the two investigator centres, with each centre having an independent sequence list for an equal number of participants. At the point of randomisation, participants will be assigned the next randomisation number in the sequence.

Confirmation that randomisation has been performed will be communicated in an un-blinded fashion to the investigator site staff, and to key members of the central research team, via emails automatically generated by the randomisation website. A researcher (usually the same researcher who conducted the baseline interview) will visit the participant in prison to deliver a letter informing the participant of the randomisation outcome. The researcher will go through the letter with the participant, ensuring that they understand their grouping and when they will be seen next and by whom (researcher or practitioner).

The Intervention

The intervention is designed to engage with individuals with common mental health problems who are close to release, developing a pathway of care in preparation for release and resettlement in the community**.** The intervention will be delivered in prison between 4 -16 weeks pre-release and for up to 20 weeks post-release. Providing they are still willing to engage, all participants will receive the intervention for eight weeks post-release. However, for those who need further support the intervention can continue for an additional 12 weeks, although at a lower intensity. This flexible approach to the length of the intervention followed on from our pilot work which indicated that whilst for many participants 2-3 months was sufficient, others required support from the practitioner for a longer period.

Engager is a manualised, person-centred intervention aiming to address mental health needs as well as to support wider issues such as accommodation, education, social relationships and money management. It was developed by bringing together evidence from a realist review,[42] focus groups, case studies and a formative process evaluation. It will be delivered by experienced support workers and supervisor team leaders with experience of therapy. A mentalisation-informed approach underpins all elements of the intervention. Use of existing practitioner skills (e.g. those used in coaching, solution-focused therapy, behavioural activation, Cognitive Behavioural Therapy) is also key to intervention delivery.

For peer review only - http://bmjopen.bmj.com/site/about/guidelines.xhtml

At pre-release stage, practitioner and participant will develop a shared understanding of the participant's needs and goals, recognising the links between emotion, thinking, behaviour and social outcomes. A goal attainment plan will be developed and followed, including liaison with relevant agencies and the participant's social networks. Engagement will be maintained throughout the pre-release period; when required, all-day support will be given on release day.

Following release, the practitioner will provide support for the participant to re-enter the community and engage with services. They will continue to work with the participant and any relevant organisations to help them achieve their goals, while encouraging the participant to take responsibility for self-care. The practitioner will also prepare the participant for the end of the intervention, while liaising with relevant community organisations regarding continuity of care.

Control group

Individuals in the control group will receive care as usual. In prison they will be able to access primary care, mental health and substance misuse services, as would usually occur. They will also receive support from criminal justice and any other third-sector organisations as standard. Their use of health, criminal justice, and third-sector services will be recorded by means of an adapted Client Service Receipt Inventory and medication usage will also be collected from prison medical records and via participant self-report in the community.

Contamination

There is unlikely to be significant contamination between the intervention and control arms of the study, although it is theoretically possible for: trainers to train practitioners elsewhere, practitioners to pass on skills and working practices to those treating control individuals, intervention materials to influence practice for control individuals, offenders to influence each other. However the risk of contamination is considered low, primarily because there is no alternative funded pathway for delivery of the substantive components of the intervention for those in the control arm. Engager practitioners form a separate team in prison and while other practitioners are informed about the intervention, i) they are not trained in the detail, ii) they tend not to have contact with our participants who are selected for the study using case finding iii) they don't have governance arrangements in place to follow individuals into the community. Cluster randomisation to prevent contamination would have been theoretically possible by randomising at a prison level, but practically not feasible because prisons are clustered together in localities, with one for new entrants, so each cluster would have several prisons. Additionally, the prison system can be subject to sudden and significant changes to prison procedures and entrants and it was estimated that a minimum of six clusters would be required in order to ensure balance, and this would have incurred prohibitive costs.

Outcome measures[1]

Outcome measure data will be collected at approximately 1 week pre-release and at 1, 3, and 6 months post-release from prison (see Table 2). The primary outcome point is at 6 months post-release. The primary outcome measure is change in levels of psychological distress as indicated by the CORE-OM. This is

[1] The selection of the primary and secondary outcome measures was informed by two consensus exercises and a period of field testing of a range of possible measure to establish the psychometric properties and acceptability of the measures in this population. This work will be presented in a separate article.

For peer review only - http://bmjopen.bmj.com/site/about/guidelines.xhtml

a 34-item scale comprising four domains, namely: subjective well-being; depression and anxiety symptoms; general, social and close relationship functioning; and items concerning risk of harm to self or others. Items are rated against how participants felt over the previous week, on a 5-point Likert Scale, with eight items reverse scored. CORE-OM was chosen as the most appropriate primary outcome measure at a consensus meeting following a period of pilot testing of a range of outcome measures. In particular, the CORE-OM is a reliable and well validated measure,[37] and was regarded as being quick to administer and easy to understand in pilot testing, and was considered to reflect the ultimate aim of the intervention. The primary outcome point is similar to that used in many previous prisoner resettlement studies, 3 to 6 months post release being widely regarded as a suitable follow-up period as the aim of resettlement interventions is to help people reintegrate into community life rather than to provide long-term support.[43]

Secondary outcome measures are as follows:

- Subjective met/unmet need across key outcome domains using the Camberwell Assessment of Need – Forensic Version (CAN-FOR).[44]
- Change in objective social domains (accommodation, education, employment and benefits).
- Drug and alcohol use using and adapted version of the Treatment Outcomes Profile (TOP).[45]
- Drug and alcohol subjective dependence using the Leeds Dependence Questionnaire (LDQ).[46]
- Service use using an adapted version of the Client Service Receipt Inventory (CSRI).[47] Recent evidence suggests that self-reported health service use data is valid in ex-prisoner population[48]
- Perceived helpfulness of services using the adapted version of the CSRI.
- Generic health related quality of life using the EQ-5D-5L questionnaire.[49]
- Wellbeing related quality of life using the ICEpop CAPability measure for adults (ICECAP-A) questionnaire.[50]
- Experience of care using the Inspire questionnaire (Relationship section only).[51]
- Change in trust, hope and motivation using the Intermediate Outcomes Measurement Instrument (IOMI).[52]
- Proven re-offending rates, based on data from the Police National Computer (PNC).

Due to the nature of the intervention, it will not be possible to blind participants or those delivering the intervention. Attempts to blind researchers during the pilot trial proved challenging, and were largely unsuccessful. Therefore researchers will be aware of which group participants are allocated to, and measures will be implemented to minimise and measure bias, especially for data collection on the primary outcome measure.[41] Specifically, the researchers will use a highly scripted interview for the primary outcome measure, reading each question to the participants and only deviating from this to clarify the meaning of the question if they indicate they do not understand the question.

For peer review only

Table 2: Tabulated summary of study schedule

		Screening	Baseline	Allocation	Pre-release	Post-release from prison			
TIMEPOINT		t_0	t_1		-1 wk t_2	+1 mth t_3	+3 mth[3] t_4	+6 mth t_5	+12 mth t_6
**ENROLMENT:**									
Eligibility screen		X							
Informed consent		X							
PHQ-9		X							
GAD-7		X							
PTSD-Screening Questionnaire		X							
Historical screen for past CMHPs		X							
Allocation[1]				X					
**INTERVENTIONS:**									
Intervention Group:	*Engager Intervention*		◆——	———	———	———	———	——◆	
	Usual care	◆——	———	———	———	———	———	———	——◆
Control Group:	*Usual care*	◆——	———	———	———	———	———	———	——◆
**ASSESSMENTS:**									
CORE-OM Questionnaire			X			X	X	X	
CORE-10[2]						X	X	X	
CANFOR – Short Version			X				X	X	
Adapted CSRI (including medication)			X		X		X	X	
Objective social outcomes (eg housing)			X					X	
Treatment Outcomes Profile (TOP)			X				X	X	
Leeds Dependence Questionnaire			X					X	
EQ-5D-5L Questionnaire			X				X	X	
ICE-CAP-A Questionnaire			X				X	X	
Intermediate Outcomes Measurement Instrument (IOMI)			X					X	
Standard Assessment of Personality (SAPAS)			X						
Neurodevelopmental Symptoms Rating Scale			X						
Trauma Questionnaire			X						
Contact Sheet			X		X	X			
Brief Inspire Questionnaire					X		X	X	
Police National Computer Offending Data			X						X
**SAFETY MONITORING:**									
Adverse event reporting		◆——	———	———	———	———	———	——◆	

[1] *Allocation will be performed using a web-based system provided by the CTU, usually within 2 days of completing the screening interview.*

[2] *CORE-10 will only be completed if it is not possible to complete the CORE-OM Questionnaire.*

For review only

For peer review only - http://bmjopen.bmj.com/site/about/guidelines.xhtml

Sample size

The sample size is based on the ability to detect a difference on the primary outcome only and not on the inclusion of baseline measures as covariate. On the CORE-OM, 5.0 points is the accepted Reliable Change Index in service evaluations.[53,54] In contrast, 2.5 points is held as the upper limit of what would be considered a change compatible with equivalence (Personal communication, Professor Michael Barkham) in trials comparing two interventions. Other trials using the CORE-OM for mental health interventions versus treatment as usual or waiting list controls have achieved mean between group differences in change score of between 3.5 and 7.8.[55,56] A standard deviation (SD) of 5.6 was found in the pilot trial.[41] However, larger clinical studies have reported larger SDs of approximately 7.5.[57]

Given the uncertainty, in both SD and the appropriate minimally clinically important difference (MCID) for the CORE-OM, we calculated sample sizes for different scenarios based on the range of values for these two parameters (see Table 3). This is equivalent to aiming to be able to detect a minimum effect size of 0.26 (i.e. small-to-medium).

Table 3: Sample size (for each group) based on different values of SD and MCID for the CORE-OM*

		Standard deviation (SD)		
		5.5	6.5	7.5
Change to be detected (MCID)	5.0	26	36	48
	4.5	32	44	59
	4.0	40	56	74
	3.5	52	73	97

*At 90% power and 2-sided alpha of 5%

Based on the conservative scenario of a MCID of at least 3.5 and a common SD of 7.5 we will require CORE-OM data on 97 participants in each group at 90% power and 5% alpha. Using an attrition rate of 30%, 140 participants are required per group. Follow-up rates of 63% and 55% were achieved in feasibility and pilot work.[41] However, with learning from the pilot trial, and assistance from the new Community Rehabilitation Companies (CRCs), which now supervise virtually all prison leavers for at least one year, an attrition rate of 30% or less is achievable.

Trial data collection

Trial data will be collected from participants at baseline, 1 week pre-release and at 1, 3 and 6 months post-release. Feasibility and pilot studies highlighted that this population often lead chaotic lives and are difficult to follow-up in the community.[41] To address this challenge, the research team will make multiple and sustained attempts to follow-up participants at each time point. Community follow-up interviews will be conducted in a convenient location for the participants, and where appropriate in the premises of services (e.g. National Probation Service or CRC) with which the participant is engaged. Participants will be provided with high street shopping vouchers compensating them for their time at the 3 and 6 month post-release interviews, although this does not apply to participants who have returned to prison and are interviewed there.

The pilot trial highlighted that some participants can be temporarily lost, but subsequently re-emerge (possibly engaging with community services or back in prison).[41] Follow-up data collection points will take

For peer review only - http://bmjopen.bmj.com/site/about/guidelines.xhtml

place within broad time-windows. The 1 month follow-up will take place between 14 and 60 days post-release, the 3 month follow-up will take place between 61 and 151 days post-release, and the 6 month follow-up between 152 and 244 days post-release. Where feasible, follow-up interviews will take place as close to 1, 3, and 6 months post-release time point as possible. Furthermore, if a participant misses a follow-up interview (e.g. at 3 months), they will continue to be included in the study until all follow-up time-points have lapsed (e.g. 245 days post-release), after which point those remaining out of contact will be regarded as lost. If the research team is in contact with a participant, but setting up a face-to-face interview is challenging (or if they have failed to turn up to an appointment), researchers will attempt to complete the CORE-10[ii] by telephone. However, even when the CORE-10 has been completed, researchers will endeavour to follow-up participants with a face-to-face interview.

The numbers and reasons for dropouts and losses to follow-up will be reported for each arm of the study.

Baseline and 1 week pre-release data collection

Baseline data collection will usually continue immediately after the screening interview, although additional sessions can be arranged to meet the needs of individual participants or time constraints within the prison. As outlined in Table 2, the following data will be collected at this point:

- Psychological distress using the CORE-OM;
- Subjective rating of need across health and social domains using adapted version of the Camberwell Assessment of Need;
- Healthcare, criminal justice and other service utilisation using an adapted version of the Client Service Receipt Inventory;
- Objective social outcomes (accommodation, education, employment);
- Drug and alcohol use and dependence using the Treatment Outcomes Profile and Leeds Dependence Questionnaire;
- Health related quality of life using the EQ-5D-5L;
- Wellbeing related quality of life using the ICE-CAP-A;
- Intermediate Outcomes Measurement Instrument (IOMI);
- Standardised Assessment of Personality – Abbreviate Scale;
- Neurodevelopmental Symptoms Rating Scale ;
- Experience of traumatic life events using the Trauma Questionnaire.

The questions from the standardised measures, including the primary outcome measure (CORE-OM) will be read out to participants in a precise and consistent manner, to minimise bias and overcome any literacy problems. Questions from the secondary outcome measures are incorporated into a specially constructed flexible interview, which avoids duplication of subject matter in order to reduce disengagement or irritability. Data will be recorded in the Baseline Care Report Form.

In addition to the baseline data collection, the researcher will complete a contact sheet for each participant. This will include contact numbers and addresses provided by the participant, as well as a list of services they are likely to be in contact with post-release. This sheet will be completed in collaboration with the participant, and they will sign the form to consent that the research team can contact them via the relevant services.

The researchers will meet with the participant again within the week prior to their release. The service use table from the adapted Client Service Receipt Inventory, to collect information on services the participant

For peer review only - http://bmjopen.bmj.com/site/about/guidelines.xhtml

has seen since the baseline data collection and the Brief Inspire Questionnaire will be completed to measure the participant's experience of these services. The researcher will also update contact details for the participant.

Information regarding medication prescribed in the 3 months before prison release, and any chronic medical conditions or acute conditions in the previous 12 months, will be collected from the prison healthcare records system. Summary data regarding offence history and number of previous custodial sentences will also be collected from prison records.

Follow-up data collection

At all follow-up meetings, the researcher will remind the participant of the information sheet and consent, drawing attention to data confidentiality and instances of disclosure where the researcher would need to breach confidentiality.

At approximately 1 month post-release, the researcher will contact the participant. This follow-up can be completed by phone, but preferably face-to-face to support continued engagement. The researcher will read aloud to the participant and record responses to the CORE-OM. These data will be used in analysis, but the main objective of the meeting is sustained engagement, and planning further contact.

The 3- and 6-month follow-ups will take place between 61-151 days and 152-244 days post-release respectively, although researchers will endeavour to complete data collection close to the 3 month (90 day) and 6 month (182 day) point. At each time point, participants will be read the questions from the measures listed in Table 2.

Economic Evaluation

The cost-effectiveness of the intervention to increase engagement and access to services, and to improve mental health outcomes will be assessed. This will be compared with service access and support as usual, using the economic model developed in the pilot phase, populated with the trial outcomes and resource use data up to 6-months post-release from prison. It will be conducted from a public sector perspective, initially with the same time horizon as the RCT, and primarily using a cost-consequence approach. Within the cost-consequence approach the estimated incremental costs will be compared with:

- The number of people provided with the service/intervention;
- The incremental differences in the main RCT self-reported health outcomes – CORE scores, and EQ-5D-5L and ICE-CAP social preference weights;
- Incremental differences in the number of ex-prisoners who: have resettled; are in employment; have no proven re-offending; are not homeless;

  Estimated lifetime gains in Quality-Adjusted Life-Years (QALYs) – Improvements in lifetime gains will be linked with short-term gains seen in the trial and will be associated with social inclusion outcomes such as effective resettlement, increased employment, or reduced re-offending rates. Sustained improvements in these will be modelled based on evidence from the literature. We will test the impact of differing the duration of the persistence of any short-term gains on QALYs (and associated costs) if there is no evidence in the literature.
- The cost of providing the intervention will be based on a combination of process of care data collection and intervention practitioner care records and diaries (bottom-up costing approach), and the total costs of service provision (top-down costing). Both deterministic and probabilistic

For peer review only - http://bmjopen.bmj.com/site/about/guidelines.xhtml

sensitivity analysis will be conducted to explore uncertainty in the model assumptions and parameters, with exploration of key sources of structural uncertainty where feasible.

The analyses will be conducted according to current guidance from the International Society for Pharmacoeconomics and Outcomes Research (ISPOR) on best practice for conducting trial-based economic evaluation.[58] Consistent with the analytical approach used in the statistical analysis of the effectiveness outcomes of the RCT where possible; the cost-effectiveness analysis will be reported in accordance with the Consolidated Health Economic Evaluation Reporting Standards.[59]

Process Evaluation

The Process Evaluation will be conducted in parallel with the trial and will adopt a mixed methods, realist informed, approach.[60] During the development and piloting of the Engager intervention we produced and refined a theoretically informed, and evidence based, logic model of the ways in which the intervention was understood to work,[61] which we intend to test in the process evaluation. The logic model included the core components of the intervention that the practitioners were asked to deliver, the key mechanisms of impact (i.e. how what the practitioners were doing was understood to produce the desired outcomes), and the anticipated outcomes.[62]

Process Evaluation Specific Objectives:

a) To determine the degree to which the core components of the intervention were delivered and the key mechanisms of the intervention occurred;
b) To evaluate the extent to which the core components and key mechanisms of the intervention produced the intended outcomes;
c) To explore any unintended consequences of delivering the intervention;
d) To identify aspects of the intervention and delivery that could be improved;
e) To identify any aspects of intervention delivery that require additional input from practitioner teams when the research team is no longer in place;
f) To develop an understanding of how to deliver the intervention in real world settings (training, supervision, meta-supervision).

Data Collection

The data collection methods were developed, and refined for acceptability, in the pilot trial Formative Process Evaluation and include:

- Intervention components checklist to measure fidelity to the intervention.
- Semi-structured interviews, with a purposively selected sub-sample of participants, some on one occasion and some at regular intervals throughout their participation in the trial;
- Semi-structured interviews with Engager practitioners and supervisors throughout the trial;
- Semi-structured interviews with other practitioners, and team leaders, in other services about their perceptions of, and interactions with, The Engager practitioners, participants and the intervention;
- Semi-structured interviews with family/partners/friends of participants receiving the Engager intervention;
- Audio-recordings of practitioner group supervision sessions;
- Audio-recordings of selected practitioner-participant interactions;
- Engager practitioner records and notes;

For peer review only - http://bmjopen.bmj.com/site/about/guidelines.xhtml

- Quantitative outcome measures, contained within the CRF, and also being used as part of the main trial outcomes
- Ethnographic field notes recorded by the Process Evaluation researchers.

Data Analysis:
The framework analysis methodology, which we developed and applied in the Formative Process Evaluation, will be utilised and extended to collate and interrogate the Process Evaluation data.[63] The deductive components of the framework will be informed by the logic model's key mechanisms of impact; that is the ways in which we understand the intervention to be working. Inductive components of the framework will be surfaces as part of the analytical process. At the end of this analytical process, the logic model of the key mechanisms of impact of the intervention will be revised.

The Process Evaluation researchers will be distinct from the researchers collecting outcome measures. They will contribute to the qualitative, and therefore more subjective, data collection and the overall analysis. A 'critical friend' researcher, external to the outcome measure and delivery teams, will facilitate the Process Evaluation researchers' opportunity to self-reflexively explore how their presence affects their data collection and experiences in the field which may influence their analytical processes.[62] When the Process Evaluation data and analysis can contribute to refining ongoing fidelity to the Engager model, it will be fed back directly to the intervention delivery team. When the Process Evaluation data and analysis concerns the outcomes of interest, the data will be shared after the trial database has been locked down and initial statistical analyses have been carried out.

If the main trial does not demonstrate that the intervention is effective, additional analysis of the qualitative data will be conducted using thematic methods to explore possible explanations for this,[64] and to glean any additional learning that may have application to other studies with socially marginalised populations and/or those with mental health needs.

Serious Adverse Events

Non-serious adverse events will not be recorded. Serious Adverse Events will be recorded and reported. Any SAEs deemed to have a causal relationship to trial participation with be reported to the Sponsor within 24 hours of the Chief Investigator being informed.

Study Timeline

Study start date – 14th January 2016
Trial Registration date – 4th February 2016[iii]
Projected end date for recruitment – 30th September 2017
Projected end date for 6 month follow-up data – 31st July 2018
Projected initial analysis of primary outcome data – 30th November 2018
Projected final report date – 31st October 2019
Current Status - recruiting

Data Management and Statistical analysis plan

All data will be treated confidentially and stored securely and anonymously. CRFs will be checked and signed at the research sites by a member of the research team before being sent to the PenCTU for double-data entry on to a password-protected database. All forms and data will be tracked using a web-based trial

For peer review only - http://bmjopen.bmj.com/site/about/guidelines.xhtml

management system. Double-entered data will be compared for discrepancies and discrepant data will be verified using the original paper data sheets.

All quantitative data analyses will be conducted and reported in accordance with Consolidated Standards of Reporting Trials (CONSORT) recommendations. We will closely monitor the process of data collection during the trial providing flow diagrams summarising, by group, the numbers approached, recruited, randomised, followed-up/lost to follow up, and outcome completion.

Primary analyses will be conducted on an intention-to-treat basis (i.e. according to randomised group), and compare primary and secondary outcomes at 6-month follow up between randomised groups on those with complete data sets. Outcomes will be compared using linear regression based methods, adjusting for baseline outcome scores and stratification variables (e.g. investigation centre). Where necessary, outcomes will be transformed to ensure good regression model fit. A secondary analysis will compare primary and secondary outcomes between groups at all follow-up time points using a repeated measures approach. Reasons for missing data (including loss to follow-up and participant drop-out) will be documented, and the baseline characteristics of those with and without missing data compared. Using different assumptions for missing data, we will undertake sensitivity analyses using various imputation models, comparing between group results to the completers primary analysis. We shall also explore the possibility of conducting secondary per protocol between group comparisons. If possible this will be based on a pre-defined minimum level of intervention receivership, and using Complier Average Causal Effect analysis methods. The analyst will be blinded to group allocation and the analysis will be undertaken using STATA v.14.2.

A detailed statistical analysis plan will be prepared before any data analysis is conducted. The statistical analysis plan will be agreed with the Trial Steering Committee.

Trial management and independent committees

Members of the research team directly involved with the day-to-day running of the trial will meet fortnightly to discuss trial progress, with additional email and telephone exchanges as required. A full Trial Management Group including health economists, statisticians, process evaluation researchers, and a sponsor representative will meet quarterly to review trial progress.

The Engager Trial Steering Committee (Chair: Professor Pamela Taylor, and three other independent members including a patient and public involvement representative) will meet 1-2 times per year to oversee the conduct of the trial, safety and ethics. The Trial Steering Committee formally agreed that given the social/psychological nature of the intervention, only limited safety monitoring would be required, and therefore an independent Data Monitoring Committee was not required.

Ethics and Dissemination

We have obtained ethical approval (Wales REC 3, reference: 15/WA/0314), National Offender Management Service (NOMS; ref: 2015-283) approval and local Trust governance approvals for each site (Devon Partnership NHS Trust, Dorset Hospital University Foundation NHS Trust and Lancashire Care NHS Foundation Trust). The study has also been adopted by the NIHR Clinical Research Network and the study Sponsor is Devon Partnership NHS Trust.

For peer review only - http://bmjopen.bmj.com/site/about/guidelines.xhtml

The trial will be conducted in accordance with the ethical principles outlined in the Declaration of Helsinki, and those consistent with GCP. The Trial Steering Committee will ensure adherence to these guidelines. Any amendments to the protocol will be submitted for ethical approval as appropriate.

Findings will be published in peer-reviewed journals and presented at local, national, and international conferences to publicise and explain the research to key audiences. A final report will be submitted to the National Institute for Health Research.

References

1. F.G. Kouyoumdjian, K.E. McIsaac, J. Liauw, et al. A systematic review of randomized controlled trial or interventions to improve the health of persons during imprisonment and in the year after release. *Am J Public Health*, 2015, 105(4), e13-e33.
2. C. Brooker, J. Repper, C. Beverley, et al. Mental health services and prisoners: A review, School of Health and Related Research (ScHARR), University of Sheffield, Sheffield, 2002.
3. D. Grubin, L. Birmingham, D. Mason. The Durham Remand Study, University of Newcastle and Newcastle City Health Trust, Her Majesty's Prison Service/ Northern and Yorkshire Regional Health Authority, London, 1997.
4. M. Rotter, B. Way, M. Steinbacher, et al. Personality Disorders in Prison: Aren't They All Antisocial?, *Psychiatr Q*, 2002, 73, 1573-6709.
5. S. Fazel,J. Danesh, Serious mental disorder in 23 000 prisoners: a systematic review of 62 surveys, *Lancet*, 2002, 359:545-550.
6. N. Singleton, H. Meltzer, R. Gatward, et al. Psychiatric morbidity among prisoners in England and Wales, The Stationery Office, London, 1998.
7. Safety in Custody Statistics Bulletin, England and Wales, Deaths in Prison Custody to December 2016, Assaults and Self-harm to September 2016. London: Ministry of Justice.
8. D. Pratt, M. Piper, L. Appleby, et al. Suicide in recently released prisoners: a population-based cohort study, *Lancet*, 2006, 368:119-123.
9. R. Borschmann, E. Thomas, P. Moran, et al. Self-harm following release from prison: a prospective data linkage study. *Aust N Z J Psychiatry*, 2017, 51(3), 250-259
10. A. Howerton, R. Byng, J. Campbell, et al. Understanding help seeking behaviour among male offenders: qualitative interview study, *BMJ*, 2007, 334, 267-268.
11. R. Byng, C. Weyer Brown, R. Sheaff, at al. Care for Offenders: Continuity of Access, NIHR Service Delivery and Organisation Programme SDO Project 08/1713/210, 2012.
12. R. Walmsley, L. Howard, S. White. The National Prison Survey 1991: Main Findings. Home Office Research Study 128, HMSO, London, 1992.
13. S. Niven, J. Olagundoye. Jobs and homes: A survey of prisoners nearing release. Home Office Research Findings 173, Home Office, London, 2002.
14. S. Niven, D. Stewart. Resettlement outcomes on release from prison in 2003. Home Office Research Findings 248, Home Office, London, 2005.
15. Social Exclusion Unit. Reducing re-offending by ex-prisoners: Report by the Social Exclusion Unit, Social Exclusion Unit, London, 2002.
16. D. Stewart. The problems and needs of newly sentenced prisoners: Results from a national survey, Ministry of Justice, London, 2008.
17. P. Lattimore & C.A. Visher. The Multi-site Evaluation of SVORI: Summary and synthesis. The Multi-site Evaluation of the Serious and Violent Offender Reentry Initiative. RTI International and Washington, DC: Urban Institute, 2009.
18. L. Hamilton,S. Belenko. Effects of pre-release services on access to behavioural health treatment after release from prison. Justice Q, 2016, 33: 1080-1102.
19. D. Wallace, C. Fahmy, L. Cotton, et al. Examining the role of familial support during prison and after release on post-incarceration mental health. *Int J Offender TherComp Criminol*, 2016, 60(1): 3-20.

For peer review only - http://bmjopen.bmj.com/site/about/guidelines.xhtml

20. K. Mallik-Kane, C. Visher. Health and Prisoner Reentry: How Physical, Mental, and Substance Abuse Conditions Shape the Process of Reintegration. Washington DC: Urban Institute; 2008.
21. H. Toi, C. Mogro-Wilson. Discharge planning for offenders with co-occurring disorders: The role of collaboration, medication, and staff. *Journal of Offender Rehabilitation*, 2015, 54: 1-18
22. Ministry of Justice. Proven re-offending statistics quarterly, April 2014 to March 2015. Ministry of Justice, 2017.
23. Home Office. IOM Efficiency Toolkit Phase 2: revised unit costs of crime and multipliers, Home Office, 2011.
24. J. Steel, G. Thornicroft, L. Birmingham, et al. 'Prison Mental Health Inreach Services', *Br J Psychiatry*, 2007, 190:373-374.
25. J. Shaw, Management of released prisoners with severe and enduring mental illness: Adaptation of the Critical Time Intervention, 2005.
26. M. Williamson. Improving the health and social outcomes of people recently released from prisons in the UK: A perspective from primary care, The Sainsbury Centre for Mental Health, London, 2006.
27. E.G. Thomas, M.J. Spittal, E.B. Heffernan, et al. Trajectories of psychological distress after prison release: implications for mental health service need in ex-prisoners. *Psychol Med*, 2016, 46(3) : 611-621.
28. J. Hague, A. Cohen. The neglected majority: Developing intermediate mental health care in primary care, London, 1995.
29. Department of Health. Improving access to psychological therapies: Offenders - positive practice guide, Department of Health, London, 2009.
30. NICE. Antisocial personality disorder: Treatment, management and prevention, National Institute for Health and Clinical Excellence, London, 2009.
31. NICE. Borderline personality disorder: Treatment and management, National Institute for Health and Clinical Excellence, London, 2009.
32. G. Durcan. Criminal Justice and Mental Health: Experiences across England and Wales, Centre for Mental Health, London, 2016.
33. W. Vanderplasschen, J. Wolf, C. G. Rapp, et al. *Effectiveness of different models of case management for substance-abusing populations, JPsychoactive Drugs*, 2007, 39:81-95.
34. E. Harvey, A. Shakeshaft, K. Hetherington, et al. The efficacy of diversion and aftercare strategies for adult drug-involved offenders: a summary and methodological review of the outcome literature, *Drug Alcohol Rev*, 2007, 26:379-387.
35. S. Lewis, M. Maguire, P. Raynor, et al. 'What Works in Resettlement? Findings from Seven Pathfinders for Short-term Prisoners in England and Wales', *Criminol Crim Justice*, 2007, 7:33-53.
36. M. Maguire, K. Holloway, M. Liddle, Fet al. Evaluation of the Transitional Support Scheme, Welsh Assembly Government, Cardiff, 2010.
37. C. Evans, J. Mellor-Clark, F. Margison, et al. CORE: Clinical Outcomes in Routine Evaluation, *JMent Health,* 2000, 9(3):247-255.
38. K. Kroenke, R. L. Spitzer, J. B. Williams. The PHQ-9: validity of a brief depression severity measure, *JGen Intern Med*, 2001, 16:606-613.
39. R. L. Spitzer, K. Kroenke, J. B. W. Williams, et al. A brief measure for assessing Generalised Anxiety Disorder: The GAD-7, *ArchIntern Med*, 2006, 166:1092-1097.
40. A. Prins, P. Ouimette, R. Kimerling, et al. The primary care PTSD screen (PC-PTSD): Development and operating characteristics, *Primary Care Psychiatry*, 2003, 9:9-14.
41. C. Lennox, T. Kirkpatrick, R. S. Taylor, et al. Pilot Randomised Controlled Trial of the ENGAGER collaborative care intervention for prisoners with common mental health problems, near to and after release, *Pilot Feasibility Stud*, 2017. doi: 10.1186/s40814-017-0163-6
42. M. Pearson, S. L. Brand, C. Quinn, J. et al. Using realist review to inform intervention development: methodological illustration and conceptual platform for collaborative care in offender mental health, *Implement Sci*, 2015, 28(10):134.
43. A. Clancy, K. Hudson, M. Maguire, et al. Getting Out and Staying Out: Results of the Prisoner Resettlement Pathfinders. Bristol: Policy Press. 2006.
44. S. Thomas, M.A. Harty, J. Parrott, et al. CANFOR: Camberwell Assessment of Need-Forensic Version, Royal College of Psychiatrists, London, Gaskell, 2003.

For peer review only - http://bmjopen.bmj.com/site/about/guidelines.xhtml

45. J. Marsden, M. Farrell, C. Bradbury, et al. Development of the treatment outcomes profile, *Addiction*, 2008, 103:1450-60.
46. D. Raistrick, J. Bradshaw, G. Tober, et al. Healey. Development of the Leeds Dependence Questionnaire (LDQ): a questionnaire to measure alcohol and opiate dependence in the context of a treatment evaluation package, Addiction, 1994, 89(5):563-72.
47. J. Beecham, M. Knapp. Costing Psychiatric Interventions, in Measuring Mental Health Needs, G. Thornicroft, C. Brewin, J. Wing (Eds.), Gaskell, 1992.
48. M. Carroll, G. Sutherland, A. Kemp-Casey, et al. Agreement between self-reported healthcare service use and administrative records in a longitudinal study of adults recently released from prison. *Health & Justice*, 2016, 4:11. DOI: 10.1186/s40352-016-0042-x.
49. EuroQoL Group. *EuroQoL:* a new facility for the measurement of health-related quality of life, *Health Policy*, 1990, 16:199–208.
50. H. Al-Janabi, T. Flynn, J. Coast. Development of a self-report measure of capability wellbeing for adults: the ICECAP-A*, Quality of Life Research*, 2012, 21(1):167-176.
51. J. Williams, M. Leamy, V. Bird, et al. Development and evaluation of the INSPIRE measure of staff support for personal recovery, *Soc Psychiatry Psychiatriatr Epidemiol*, 2015, 50(5):777-86.
52. M. Maguire, E. Disley, M. Liddle, et al. Developing a toolkit to measure intermediate outcomes to reduce reoffending, London: NOMS, 2017, Ministry of Justice Analytical Series.
53. N.S. Jacobson, P. Truax. Clinical significance: A statistical approach to defining meaningful change in psychotherapy research, *J Consult Clin Psychol,* 1991, 59(1):12–19.
54. J. Connell, M. Barkham, W.B. Stiles, et al. Distribution of CORE-OM scores in a general population, clinical cut-off points and comparison with the CIS-R*, Br J Psychiatry*, 2007, 190,:69–74.
55. C. Thiels, U. Schmidt, J. Treasure, et al. Guided self-change for bulimia nervosa incorporating use of a self-care manual, *Am J Psychiatry*, 1998, 155:947 -953.
56. L.B. Nordgren, E. Hedman, J. Etienne, et al. Effectiveness and cost-effectiveness of individually tailored Internet-delivered cognitive behavior therapy for anxiety disorders in a primary care population: a randomized controlled trial, *Behav Res Ther,* 2014, 59:1–11.
57. M. Lucock, K. Padgett, R. Noble, et al. Controlled Clinical Trial of a Self-Help for Anxiety Intervention for Patients Waiting for Psychological Therapy, *Behavioural and Cognitive Psychotherapy* , 2008, 36(5):541-551.
58. S.D. Ramsey, R.J. Willke, H.A. Glick, et al. Cost-effectiveness analysis alongside clinical trials II: An ISPOR good research practices task force report. *Value Health*, 2015, 18:161-172.
59. D. Husereau, M. Drummond, S. Petrou, et al. Consolidated Health Economic Evaluation Reporting Standards (CHEERS) statement. *BMJ*, 2013; 346:f1049.
60. R. Pawson,N. Tilley. Realistic Evaluation, London, Sage Publications, 1997.
61. G. Moore, S. Audrey, M. Barker, et al. Process evaluation of complex interventions: Medical Research Council guidance, MRC Population Health Science Research Network, London, 2014.
62. P. Craig, P. Dieppe, S. Macintyre, et al. Developing and evaluating complex interventions: the new Medical Research Council guidance, *BMJ*, 2008, 337:1655.
63. J. Ritchie, L. Spencer. "Qualitative data analysis for applied policy research" in A. Bryman and R.G. Burgess (Eds.), Analysing Qualitative Data*,* London, Routledge, 1994, 173-94.
64. V. Braun, V. Clarke. "Using thematic analysis in psychology", *Qual Res Psychol*, 2006*,* 3(2):77-101.

Author's contribution

TK, CS and LC wrote the first draft of the manuscript. RB is the Chief Investigator on the NIHR grant and RST, RA, MM, MH, SM, CO, GD, AS, WH, CQ, TH, MP and JS are co-applicants on the grant and contributed

For peer review only - http://bmjopen.bmj.com/site/about/guidelines.xhtml

to the conceptualisation of the study design. CL, SLB, ASte, RT, SR-B, and RG are members of the study team that have contributed to the analysis of pilot data and development of the trial methodology. All authors provided critical evaluation of the manuscript and have given final approval of the manuscript.

Funding statement

Engager is funded by the National Institute for Health Research (NIHR) under its Programme Grant for Applied Research Programme (Grant number: RP-PG-1210-12011). This research was also supported by the National Institute for Health Research (NIHR) Collaboration for Leadership in Applied Health Research and Care South West Peninsula at the Royal Devon and Exeter NHS Foundation Trust. The views expressed are those of the author(s) and not necessarily those of the NHS, the NIHR or the Department of Health. The funder had no role in the design of this study and will not have any role during execution, analysis, interpretation of findings, or decision to submit results. The views expressed are those of the authors and not necessarily those of the NHS, the NIHR, or the Department of Health.

Competing Interests

The authors declare that they have no competing interests.

---

[i] Participants will be directly asked whether they had periods of two weeks or more in the two years before coming into prison when they experiences a problem (e.g. stress), whether this affected them functioning normally in everyday tasks, and whether they think this will be a problem again once they have been released. These three questions were then repeated for problems involving feeling down or depressed, feeling anxious or worrying a lot, having nightmares or horrible thoughts, or having panic attacks. If the participants responds yes to all three questions for any of the 5 types of problems and could provide an example of how it affected their functioning, then they met this inclusion criteria for the study.

[ii] The CORE-10 will be used when completing the measure over the phone because it is shorter. The 34-item CORE-OM was considered too long to complete over the phone with this population of participants.

[iii] The trial was retrospectively registered about three weeks after the first participant was recruited. The trial was registered on the National Institute for Health Research (NIHR) Portfolio Database on 15th December 2015, but we encountered delays in the information being transferred for trial registration. We only became aware of this shortly after we had started recruitment and the issue was quickly rectified and the trial was registered on 4th February 2016.

For peer review only - http://bmjopen.bmj.com/site/about/guidelines.xhtml

Appendix 1

PLYMOUTH UNIVERSITY PENINSULA SCHOOLS OF MEDICINE & DENTISTRY

engager healthy minds, healthy choices

MANCHESTER 1824 The University of Manchester

Information for people considering taking part in the Engager Randomised Controlled Trial

What is the purpose of the study?

We have put together a package of care that aims to help people in prison and after they are released from prison. The purpose of this study is to find out how well this package of care works.

Why have I been asked to take part?

We are interested in talking to prisoners near release who may be experiencing anxiety, distress or feeling low.

Do I have to take part?

No, it is up to you. If you would prefer not to take part then you do not have to give a reason and you will not be under any pressure to change your mind, and this decision will not affect the normal help and services you receive when leaving prison. If you do decide to take part you will be asked to sign a consent form to show you have agreed to take part. If you do decide to take part then you are free to leave the study at any time. You do not have to give a reason but any information you have already given will remain part of the research and the research team may contact you to ask if there are ways in which you think the service could be improved.

What does taking part involve?

If you agree to take part, we will first ask you some questions about how you have been feeling over recent weeks and about any problems you had before you came into prison. This should take about 20 minutes and will help us make a decision about whether the intervention is suitable for you. If the intervention is not suitable for you then we will not need to see you again and that will be the end of your participation in the study. This does not mean that your problems are more or less important than anyone else's problems; it just means that our intervention is not suitable for you.

If the intervention it suitable for you then we would like to ask you further questions about how you have been feeling recently and what sort of things are likely to be a problem for you when you get released. This should take about 1 hour and we can do it immediately after the first set of questions or we can arrange another time for me to come back and see you.

Once you have finished these questions, you will be put in either the Engager Intervention group or the Treatment As Usual group. A computer programme will randomly put you in one of the two groups. If you are not put in the Engager intervention group it does not mean that your needs are any more or less important.

Treatment as usual

If you are in the Treatment As Usual group you will be involved in the standard discharge planning within the prison, nothing different will happen to you and you will receive all the services that you would normally receive leading up to and following your release from prison.

Engager Intervention

If you are in the Engager Intervention group you will receive all the standard discharge planning that you would normally receive, but you will also work with the Engager Intervention team. While in prison they will work with you to look at what your needs might be on release from prison and develop a tailor-made package of care for you. This will include supporting you in managing your anxiety and mood, and looking at your goals and concerns about

For peer review only - http://bmjopen.bmj.com/site/about/guidelines.xhtml

release which might include housing, finances, relationships, and health and wellbeing. They will also explore any community services that might be able to help you when you are released from prison.

The Engager Intervention team will continue to work with you through the gate and for up to four months following your release to support you. They will be in contact with you between 10 and 20 times in the three months following your release from prison, depending on your needs and how well things are going for you. Some of these contacts will be face-to-face, but others may be by phone. We may want to record one or more of your meetings with the Engager Intervention team for training purposes. The Engager Intervention team will inform you at the start of any meeting that they would like to record it and seek your written permission to do so. It is entirely up to you whether you give permission for any of your meetings to be recorded. If you do not want to have any meetings recorded, this will not affect your participation in other aspects of the study.

We will ask for your permission for the Engager Intervention team to access your criminal justice and health records in order to help them develop a tailor-made package of care with you. We will also ask your permission to share the questionnaires you have just completed with them.

The Engager team may also need to share your tailor-made care plan with other organisations that will be involved in helping you.

Will I have to do anything else?

Regardless of whether you are allocated to the 'Engager Intervention' group or 'Treatment As Usual' group, a member of the research team would like to stay in touch with and meet with you to ask you some questions about how well you have been getting on. The questions will be about your health needs, use of services and how you have been feeling. This will be around a week before you are released and at 4 weeks, 3 months, 6 months, and 12 months following your release from prison. We will speak to you about this nearer the time. As a thank you for staying in touch you will receive high-street vouchers at the end of the 3, 6, and 12 months sessions, although you will only receive these if we meet you in the community and not if you are in prison at the time.

We will ask for your permission for the Engager Research team to access your criminal justice and health records in order to inform the research. Information will be collected after your session with the researcher today and then again in 12 months' time from the Police National Computer to see if any of this information has changed.

While you are in prison and after your release, you may also be invited to an interview with a researcher to discuss your thoughts about any treatment you may have received, and your experience of being involved in the study. Similarly, if you decide not to take part in the Randomised Controlled Trial you may be invited to be interviewed in order to help us to understand the reasons why you don't want to be involved. It is entirely up to you to decide whether you want to take part in further parts of the study and by agreeing to take part now you are not committing yourself to being involved in any other parts of the study.

What are the possible benefits of taking part?

We can't promise that the study will help you directly, but we hope that for those in the Engager Intervention group taking part in the study may help you to manage your anxieties and mood more effectively and improve the contact you have with services after release from prison. Findings from the research may also help to improve services for future prisoners when they are released from prison.

What are the possible risks or disadvantages of taking part?

You will be asked to give up some of your time to take part. We do not see any serious risks in taking part in the study. Occasionally some people may experience some emotional distress when they are asked to think about their experiences. If you would prefer not to answer any individual questions just say so and we will more onto the next

For peer review only - http://bmjopen.bmj.com/site/about/guidelines.xhtml

question. If you are upset you will be able to talk to the researcher about it. If you feel you require further support they will be able to tell you about other possible sources of help or advice.

What if there is a problem?

If you are in prison or under probation supervision then you should direct any requests for information, complaints, concerns and queries through the prison establishment or the probation service. If you are not in prison or under probation supervision and have a problem or concern about the way you have been approached or treated during this study then you should ask to speak to the researcher, who will do their best to answer your questions. In the event that something goes wrong and you are harmed during the research, and this is due to someone's negligence, then you may have grounds for a legal action for compensation but you may have to pay your own legal costs.

Will my taking part in the study be kept confidential?

Yes. Any information collected about you will be kept strictly confidential and will not be disclosed outside the research team without your permission. Any personal information that we collect about you, and any consent forms will be stored securely and will only be used for the purposes of the research. Transcription of audio recordings of any meetings between yourself and the Engager team will only be done by members of the research team or other individuals who have signed confidentiality agreements. Any quotations from research participants used in the project report will be anonymised and no real names will be used.

You have the right to check the accuracy of the data held about you and to correct any errors. Procedures for the handling, processing, storage and destruction of your data will be compliant with the Data Protection Act 1998. Some parts of the data collected for the study may be looked at by authorised representatives of regulatory authorities to check that the study is being correctly carried out, but this information will remain anonymous. Should this occur, all such individuals will have a duty of confidentiality to you as a research participant. The data will be securely disposed of after 5 years.

Are there any circumstances in which confidentiality would be broken?

Yes. You should be aware that the researcher has a duty to inform an appropriate person should you disclose any of the following:

a) Behaviour that is against prison rules and can be adjudicated against
b) Information that indicates a risk of harm to yourself or others or refers to a new crime committed or plans to commit a new crime
c) Undisclosed illegal acts
d) Information that raises concerns about terrorism, radicalisations, or security issues.

What will happen to the results of the study?

The results of this study may be published in a report or criminal justice or medical journal. If you would like a copy of any publication or a summary of the results, please let the researcher know. You will not be identified in any report or publication arising from the study.

Who will know if I am taking part in this study?

The research team and practitioners delivering the intervention will know you are taking part in the study. Staff from the prison service may also be aware that you are taking part in the research, but they will not have access to any information we collect during the course of the study.

For peer review only - http://bmjopen.bmj.com/site/about/guidelines.xhtml

Who has reviewed this study?

The research has been looked at by an independent group of people, called a Research Ethics Committee (REC) to protect your safety, rights, wellbeing and dignity. This study has been reviewed and given a favourable opinion by NRES Committee Wales REC 3. The research has also been reviewed and approved by the Research & Development Offices of your local NHS Trust and NOMS.

For peer review only

For peer review only - http://bmjopen.bmj.com/site/about/guidelines.xhtml

Appendix 2

CONSENT FORM

Please ***initial***

1. I confirm that I have read and understood the research information sheet ('Information for people considering taking part in the Engager Randomised Controlled Trial') dated 24/03/2017, Version Five, and have been given the opportunity to ask questions. ☐
2. I understand that my participation is voluntary and that I am free to withdraw at any time, without giving any reason and without my legal rights being affected. ☐
3. I understand that if I withdraw from the study I will stop receiving the intervention, but this will not affect any other aspects of my medical care. ☐
4. I understand that whilst I participate in the RCT, the researcher has a duty to inform prison staff should I disclose:
   a) Behaviour that is against prison rules and can be adjudicated against
   b) Information that either indicates a risk or harm to yourself or others or refers to a new crime committed or plans to commit a new crime ☐
   c) Undisclosed illegal acts
   d) Information that raises concerns about terrorist, radicalisations, or security issues.
5. I understand that I will be assigned to the intervention group or treatment as usual group at random, and I won't be able to choose which group I am in. ☐
6. I understand that I may be asked for permission to digitally record one or more of my meetings with the Engager team, but I am under no obligation to give permission. ☐
7. I give permission for the research team to look at my criminal justice and health records for the purpose of informing the research. ☐
8. I give the research team permission to request my criminal justice records from the Police National Computer in 12 months' time ☐
9. I agree to take part in the above study. ☐
10. I do not wish to take part in the study but agree to be contacted to discuss my reasons for not wanting to be involved. ☐
11. I understand that I may be contacted by the research team in the future, but am under no obligation to participate in any further research. ☐

Sign below for participant completed consent form

Name of participant	Date	Signature
Name of researcher	Date	Signature

p.t.o. for researcher completed consent forms

For peer review only

For peer review only - http://bmjopen.bmj.com/site/about/guidelines.xhtml

Appendix 2

PLYMOUTH UNIVERSITY PENINSULA SCHOOLS OF MEDICINE & DENTISTRY

engager
healthy minds, healthy choices

MANCHESTER 1824
The University of Manchester

Sign below for researcher completed consent form

"I [researcher name] have read this form of consent to [participant name] because [participant name] is not able to read this informed consent document.

I have asked [participant name] to make his mark to confirm that he has understood the informed consent document"

Name of participant	Date	Signature
Name of researcher	Date	Signature

*1 copy for participant: 1 copy for researcher

Principal Investigator: Professor Richard Byng, Plymouth University Peninsula Schools of Medicine and Dentistry

For peer review only

For peer review only - http://bmjopen.bmj.com/site/about/guidelines.xhtml

SPIRIT

STANDARD PROTOCOL ITEMS: RECOMMENDATIONS FOR INTERVENTIONAL TRIALS

SPIRIT 2013 Checklist: Recommended items to address in a clinical trial protocol and related documents*

**Section/item**	**Item No**	**Description**	**Page Number on which item is reported**
**Administrative information**			
Title	1	Descriptive title identifying the study design, population, interventions, and, if applicable, trial acronym	1
Trial registration	2a	Trial identifier and registry name. If not yet registered, name of intended registry	6
	2b	All items from the World Health Organization Trial Registration Data Set	Various
Protocol version	3	Date and version identifier	
Funding	4	Sources and types of financial, material, and other support	21
Roles and responsibilitie s	5a	Names, affiliations, and roles of protocol contributors	1,2
	5b	Name and contact information for the trial sponsor	17
	5c	Role of study sponsor and funders, if any, in study design; collection, management, analysis, and interpretation of data; writing of the report; and the decision to submit the report for publication, including whether they will have ultimate authority over any of these activities	21
	5d	Composition, roles, and responsibilities of the coordinating centre, steering committee, endpoint adjudication committee, data management team, and other individuals or groups overseeing the trial, if applicable (see Item 21a for data monitoring committee)	17
**Introduction**			

For peer review only

For peer review only - http://bmjopen.bmj.com/site/about/guidelines.xhtml

Background and rationale	6a	Description of research question and justification for undertaking the trial, including summary of relevant studies (published and unpublished) examining benefits and harms for each intervention	4,5
	6b	Explanation for choice of comparators	10
Objectives	7	Specific objectives or hypotheses	5,6
Trial design	8	Description of trial design including type of trial (eg, parallel group, crossover, factorial, single group), allocation ratio, and framework (eg, superiority, equivalence, noninferiority, exploratory)	6
**Methods: Participants, interventions, and outcomes**			
Study setting	9	Description of study settings (eg, community clinic, academic hospital) and list of countries where data will be collected. Reference to where list of study sites can be obtained	6
Eligibility criteria	10	Inclusion and exclusion criteria for participants. If applicable, eligibility criteria for study centres and individuals who will perform the interventions (eg, surgeons, psychotherapists)	7
Interventions	11a	Interventions for each group with sufficient detail to allow replication, including how and when they will be administered	8,9
	11b	Criteria for discontinuing or modifying allocated interventions for a given trial participant (eg, drug dose change in response to harms, participant request, or improving/worsening disease)	8
	11c	Strategies to improve adherence to intervention protocols, and any procedures for monitoring adherence (eg, drug tablet return, laboratory tests)	12,13
	11d	Relevant concomitant care and interventions that are permitted or prohibited during the trial	8
Outcomes	12	Primary, secondary, and other outcomes, including the specific measurement variable (eg, systolic blood pressure), analysis metric (eg, change from baseline, final value, time to event), method of aggregation (eg, median, proportion), and time point for each outcome. Explanation of the clinical relevance of chosen efficacy and harm outcomes is strongly recommended	9-11

For peer review only - http://bmjopen.bmj.com/site/about/guidelines.xhtml

Participant timeline	13	Time schedule of enrolment, interventions (including any run-ins and washouts), assessments, and visits for participants. A schematic diagram is highly recommended (see Figure)	11-14
Sample size	14	Estimated number of participants needed to achieve study objectives and how it was determined, including clinical and statistical assumptions supporting any sample size calculations	12
Recruitment	15	Strategies for achieving adequate participant enrolment to reach target sample size	12
**Methods: Assignment of interventions (for controlled trials)**			
Allocation:			
Sequence generation	16a	Method of generating the allocation sequence (eg, computer-generated random numbers), and list of any factors for stratification. To reduce predictability of a random sequence, details of any planned restriction (eg, blocking) should be provided in a separate document that is unavailable to those who enrol participants or assign interventions	8
Allocation concealment mechanism	16b	Mechanism of implementing the allocation sequence (eg, central telephone; sequentially numbered, opaque, sealed envelopes), describing any steps to conceal the sequence until interventions are assigned	8
Implementation	16c	Who will generate the allocation sequence, who will enrol participants, and who will assign participants to interventions	8
Blinding (masking)	17a	Who will be blinded after assignment to interventions (eg, trial participants, care providers, outcome assessors, data analysts), and how	10
	17b	If blinded, circumstances under which unblinding is permissible, and procedure for revealing a participant's allocated intervention during the trial	10
**Methods: Data collection, management, and analysis**			

Data collection methods	18a	Plans for assessment and collection of outcome, baseline, and other trial data, including any related processes to promote data quality (eg, duplicate measurements, training of assessors) and a description of study instruments (eg, questionnaires, laboratory tests) along with their reliability and validity, if known. Reference to where data collection forms can be found, if not in the protocol	12-14
	18b	Plans to promote participant retention and complete follow-up, including list of any outcome data to be collected for participants who discontinue or deviate from intervention protocols	12-14
Data management	19	Plans for data entry, coding, security, and storage, including any related processes to promote data quality (eg, double data entry; range checks for data values). Reference to where details of data management procedures can be found, if not in the protocol	16
Statistical methods	20a	Statistical methods for analysing primary and secondary outcomes. Reference to where other details of the statistical analysis plan can be found, if not in the protocol	16-17
	20b	Methods for any additional analyses (eg, subgroup and adjusted analyses)	16
	20c	Definition of analysis population relating to protocol non-adherence (eg, as randomised analysis), and any statistical methods to handle missing data (eg, multiple imputation)	16-17
**Methods: Monitoring**			
Data monitoring	21a	Composition of data monitoring committee (DMC); summary of its role and reporting structure; statement of whether it is independent from the sponsor and competing interests; and reference to where further details about its charter can be found, if not in the protocol. Alternatively, an explanation of why a DMC is not needed	N/A see page 17
	21b	Description of any interim analyses and stopping guidelines, including who will have access to these interim results and make the final decision to terminate the trial	17

For peer review only - http://bmjopen.bmj.com/site/about/guidelines.xhtml

Harms	22	Plans for collecting, assessing, reporting, and managing solicited and spontaneously reported adverse events and other unintended effects of trial interventions or trial conduct	16
Auditing	23	Frequency and procedures for auditing trial conduct, if any, and whether the process will be independent from investigators and the sponsor	17
**Ethics and dissemination**			
Research ethics approval	24	Plans for seeking research ethics committee/institutional review board (REC/IRB) approval	17
Protocol amendments	25	Plans for communicating important protocol modifications (eg, changes to eligibility criteria, outcomes, analyses) to relevant parties (eg, investigators, REC/IRBs, trial participants, trial registries, journals, regulators)	17
Consent or assent	26a	Who will obtain informed consent or assent from potential trial participants or authorised surrogates, and how (see Item 32)	7
	26b	Additional consent provisions for collection and use of participant data and biological specimens in ancillary studies, if applicable	N/A
Confidentiality	27	How personal information about potential and enrolled participants will be collected, shared, and maintained in order to protect confidentiality before, during, and after the trial	16
Declaration of interests	28	Financial and other competing interests for principal investigators for the overall trial and each study site	21
Access to data	29	Statement of who will have access to the final trial dataset, and disclosure of contractual agreements that limit such access for investigators	Unknown
Ancillary and post-trial care	30	Provisions, if any, for ancillary and post-trial care, and for compensation to those who suffer harm from trial participation	None N/A
Dissemination policy	31a	Plans for investigators and sponsor to communicate trial results to participants, healthcare professionals, the public, and other relevant groups (eg, via publication, reporting in results databases, or other data sharing arrangements), including any publication restrictions	17

For peer review only

	31b	Authorship eligibility guidelines and any intended use of professional writers	N/A
	31c	Plans, if any, for granting public access to the full protocol, participant-level dataset, and statistical code	N/A
**Appendices**			
Informed consent materials	32	Model consent form and other related documentation given to participants and authorised surrogates	See attached documentation
Biological specimens	33	Plans for collection, laboratory evaluation, and storage of biological specimens for genetic or molecular analysis in the current trial and for future use in ancillary studies, if applicable	N/A

*It is strongly recommended that this checklist be read in conjunction with the SPIRIT 2013 Explanation & Elaboration for important clarification on the items. Amendments to the protocol should be tracked and dated. The SPIRIT checklist is copyrighted by the SPIRIT Group under the Creative Commons "Attribution-NonCommercial-NoDerivs 3.0 Unported" license.

For peer review only
